# Polymeric piezoelectric accelerometers with high sensitivity, broad bandwidth, and low noise density for organic electronics and wearable microsystems

**DOI:** 10.1038/s41378-024-00704-6

**Published:** 2024-05-15

**Authors:** Chang Ge, Edmond Cretu

**Affiliations:** The Department of Electrical and Computer Engineering, The University of British Columbia, Vancouver, BC Canada

**Keywords:** Electrical and electronic engineering, Sensors

## Abstract

Piezoelectric accelerometers excel in vibration sensing. In the emerging trend of fully organic electronic microsystems, polymeric piezoelectric accelerometers can be used as vital front-end components to capture dynamic signals, such as vocal vibrations in wearable speaking assistants for those with speaking difficulties. However, high-performance polymeric piezoelectric accelerometers suitable for such applications are rare. Piezoelectric organic compounds such as PVDF have inferior properties to their inorganic counterparts such as PZT. Consequently, most existing polymeric piezoelectric accelerometers have very unbalanced performance metrics. They often sacrifice resonance frequency and bandwidth for a flat-band sensitivity comparable to those of PZT-based accelerometers, leading to increased noise density and limited application potentials. In this study, a new polymeric piezoelectric accelerometer design to overcome the material limitations of PVDF is introduced. This new design aims to simultaneously achieve high sensitivity, broad bandwidth, and low noise. Five samples were manufactured and characterized, demonstrating an average sensitivity of 29.45 pC/g within a ± 10 g input range, a 5% flat band of 160 Hz, and an in-band noise density of 1.4 µg/$\sqrt{{Hz}}$. These results surpass those of many PZT-based piezoelectric accelerometers, showing the feasibility of achieving comprehensively high performance in polymeric piezoelectric accelerometers to increase their potential in novel applications such as organic microsystems.

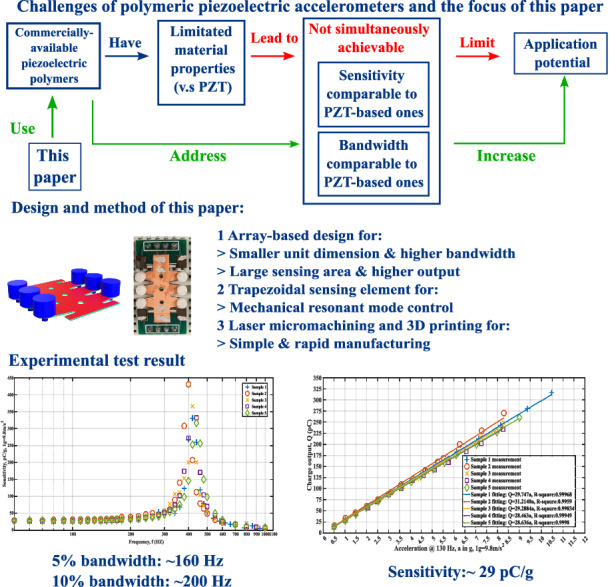

## Introduction

Recent rapid progress in the research of organic functional materials has significantly promoted the innovations of organic electronics and polymeric microsystems^1–3^. A primary research topic in this respect is developing wearable electronics and intelligent systems. For these novel microsystems, polymeric sensors are vital front-end components that convert mechanical signals from the surroundings into electrical signals to be processed by organic circuits, augmenting the cognitive capability of a human being.

Evolving from their silicon or inorganic microelectromechanical systems (MEMS) counterparts, polymeric sensors for organic electronics and microsystems use similar working principles and structural designs. The differences in the material properties have led to advantages and disadvantages in terms of microfabrication and performance. On the one hand, many new polymer processing methods simplify the sensor manufacturing process by omitting the traditional micromachining methodology based on cycles of material deposition, lithography, and anisotropic etching. As typical examples in this respect, many studies have combined direct processing methods, such as roll-to-roll adhesive lamination^4,5^, laser micromachining^6^, aerosol jet printing^7,8^, and 3D printing^9^, to manufacture polymeric sensors for wearable microsystems. On the other hand, most of these direct machining methods cannot compete with classic micromachining techniques for resolution and throughput. Moreover, the properties of many organic functional materials are not comparable to those of their inorganic counterparts. Correspondingly, polymeric sensors manufactured by these novel techniques have larger dimensions than their inorganic counterparts fabricated by classic micromachining methods to obtain specific competitive performance metrics^10,11^. For some polymeric sensors, their larger size may negatively impact their performance and application potential. One typical example of this perspective is the polymeric piezoelectric accelerometers.

Piezoelectric accelerometers provide unique advantages in vibration sensing, such as fast response and broad input range^12,13^. The amount of charge generated by piezoelectric coupling is proportional to the area integral of the product between the reaction stress and the piezoelectric coupling coefficient^14^; therefore, existing piezoelectric accelerometers typically utilize cantilever-based structures and lead zirconate titanate (PZT)^15–17^ for high performance to play crucial roles in automotive systems^18^, consumer electronics^19^, image stabilization^20^, implantable hearing aid devices^21,22^, and vibration monitoring for apparatuses. In emerging organic electronics and polymeric wearable microsystems, as indicated by existing studies^23,24^, polymeric piezoelectric accelerometers can serve as wearable vibration collectors in many critical applications; these include vocal assistance systems for those with speaking difficulties, such as amyotrophic lateral sclerosis (ALS) victims. The vocal system vibrates between 85 and 116 Hz (male) or between 85 and 210 Hz (female) when a person speaks^25^. Although this frequency range can be considered the ideal frequency range for vibration sensing using a piezoelectric accelerometer, it is beyond the capability of most existing polymeric piezoelectric accelerometers. Piezoelectric polymers undeniably lack the mechanical and piezoelectric properties of PZT^26^. Consequently, most polymeric accelerometers^27–32^ have resorted to lower resonant frequencies to rival PZT-based accelerometers in terms of sensitivity. However, this design strategy leads to reduced bandwidth and increased noise density^33^. Most existing polymeric piezoelectric accelerometers^27–32^ have a flat bandwidth below 100 Hz, preventing them from fulfilling the aforementioned vital role in wearable polymeric vocal assistants.

For example, we previously developed a polymeric piezoelectric accelerometer using PVDF^29^ due to its advantageous material properties and experimentally validated its robustness; PVDF has a higher piezoelectric coefficient^26^ and better mechanical, chemical, and thermal stability^34^ than many polymeric piezoelectric materials based on nanomaterial dopants. Some studies^35,36^ have used PVDF thin films and lamination processes for assembly to develop energy harvesters for wearable electronics in which PVDF-based structures can maintain relatively consistent output after undergoing high-magnitude mechanical stress in hundreds or even thousands of cycles. The mechanical load of the bending and stretching tests in these studies^35,36^ is more significant than the vocal vibration. Hence, to a certain extent, the consistency reported in these studies^35,36^ supports the suitability of utilizing PVDF for polymeric piezoelectric accelerometers used as vocal vibration sensors in wearable microsystems with chronic performance stability. Our previous PVDF-based accelerometer had a sensitivity of 21.82 pC/g. Although this flat-band sensitivity was comparable to those of several recent PZT-based accelerometers^15,16^, our previous polymeric piezoelectric accelerometers only have a limited bandwidth of 58.5 Hz.

In this study, the challenge pertaining to polymeric piezoelectric accelerometers is addressed by a PVDF-based polymeric piezoelectric accelerometer design with higher sensitivity, broader bandwidth, and lower noise levels than existing polymer piezoelectric accelerometers. An issue potentially associated with the high structural flexibility of existing polymeric piezoelectric accelerometers was discovered. By addressing this issue during structure design in a mathematically controlled manner, the PVDF-based polymeric piezoelectric accelerometer presented in this study is experimentally validated to have the highest sensitivity, broadest flat bandwidth, and lowest noise level among polymeric piezoelectric accelerometers to the best of our knowledge, and it even surpasses several PZT-based piezoelectric accelerometers. These experimentally validated high performances for the polymeric piezoelectric accelerometers significantly increase their application potentials as front-end sensors in wearable microsystems to assist patients with vocal disability.

In the following sections, first, we present the design of the PVDF-based accelerometers. After briefly introducing our manufactured samples, we provide comprehensive experimental characterizations, including mechanical resonance measurements, vibrational behavior analysis, frequency response to accelerations, flat-band sensitivity to accelerations, and noise-level assessments. In the discussion section, we thoroughly compare the characterization results from our five PVDF-based polymeric piezoelectric accelerometers, their PZT-based counterparts, and other polymeric piezoelectric accelerometers. Following the discussion, we provide a brief overview of the microfabrication process and experimental methods before concluding with a summary of our study’s achievements.

## Results

### Design of the PVDF-based piezoelectric accelerometers with high sensitivity and resonant frequency

**Fig. 1:**
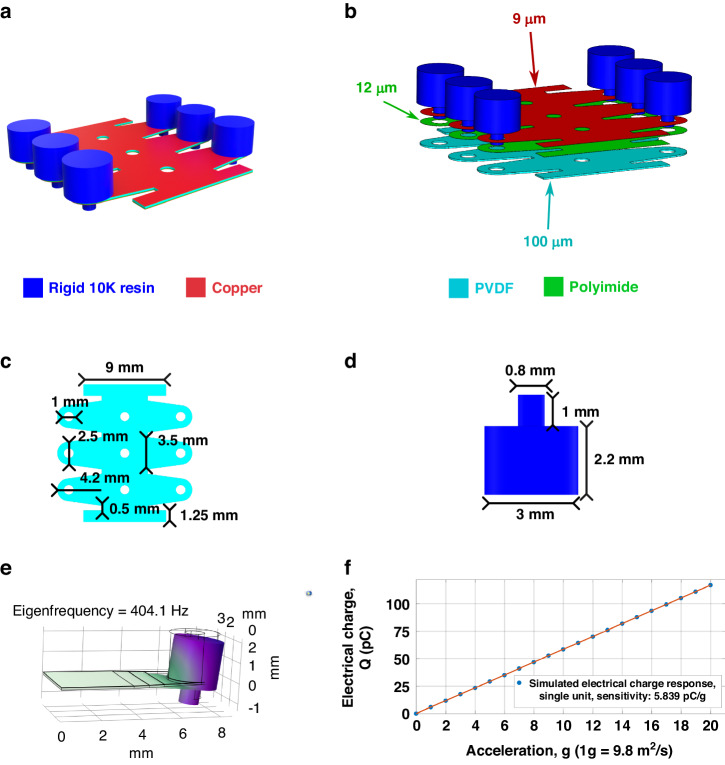
PVDF-based polymeric piezoelectric accelerometer design using tapered cantilevers as the sensing units. **a** 3D view of a single device; **b** expanded view of a single device with a labeled thickness; **c** planar dimensions of the polymeric layers; **d** cross-sectional dimension of the inertial mass; **e** FEA-simulated fundamental resonant frequency; **f** FEA-simulated flat-band sensitivity

Piezoelectric accelerometers have two primary sources of intrinsic noise: thermomechanical and thermoelectrical; the latter one is more prominent^33^. Moreover, thermoelectrical noise is inversely related to the bandwidth. Due to this unique characteristic of piezoelectric accelerometers, the three objectives of this study are theoretically equivalent to implementing a polymeric piezoelectric accelerometer with higher sensitivity and resonant frequency than existing ones. Figure 1 shows the details of the new design.

As shown in Fig. 1a, b, the new design is based on the off-the-shelf polymer thin films and 3D-printed components. Each component is separately manufactured and adhesively laminated together. Supported by existing studies about polymeric tactile sensors for organic electrical microsystems or wearable intelligent systems^37,38^, this device manufacturing strategy can simplify the fabrication process of polymer transducers through the utilization of direct micromachining techniques. The footprint of a single accelerometer in Fig. 1 is set to 15 mm by 15 mm. This dimensional limit is based on the typical size summarized in recent studies on film-based polymer sensors for fully organic or wearable systems^9,39^. A footprint of a similar size can ensure that the polymer sensors have sufficient contact with the human body for signal collection during potential practical applications^39^.

Figure 1 shows that a single polymeric piezoelectric accelerometer features an array of six identical cantilever sensing units that are electrically connected in parallel, with their piezoelectric charge responses summed for the output. Compared with other existing polymeric piezoelectric accelerometers^27,28,30–32^, this array-based design enable the significant reduction of the planar dimensions of the individual units, achieving miniaturization for a high resonant frequency while maintaining a comparable total effective sensing area to obtain high sensitivity. The number of sensing units is set to six, the same as in our previous design^29^. The primary purpose is to simplify the design process, focusing only on optimizing a single sensing unit.

Inspired by existing studies on controlling cantilever vibrational behaviors^40–44^, each sensing unit in Fig. 1 uses a trapezoid cantilever, aiming to effectively suppress higher-order harmonic resonant modes. Although varying the cantilever thickness can also fulfill a similar goal, the fabrication process of such a structure could be more complex. Hence, we adopt trapezoidal cantilevers to curb higher-order harmonic modes. The necessity of this geometric design is supported by the mechanical resonance analysis of our previous design of PVDF-based polymer accelerometers^29^. The sensing structure of this design has dimensions of 2 mm by 7.5 mm, leading to greater structural flexibility than that of the design in Fig. 1. The mechanical resonance analysis revealed that the sensing structures based on the rectangular cantilevers promoted higher-order harmonic resonant modes that potentially caused the actual sensitivity to be significantly lower than its simulated theoretical ceiling. Details of the experiment are available in Supplement Material A: Mechanical characterization of old samples.

The dimensions of each trapezoid cantilever sensing unit in Fig. 1b, c are also numerically optimized to fit the 15 mm-by-15 mm area limit set up at the beginning of the design. To control the flat-band sensitivity while adjusting the dimensional design and resonant frequency, we establish a mathematical model of the sensitivity, mechanical resonance, and dimensional design parameters. Please refer to Supplemental Material B: Mathematical model derivation. Here, we only show the final model:

1 $$s=\frac{{Q}_{piezo}}{{a}_{input}}\propto {d}_{31}E\frac{1}{{\omega }_{0}^{2}}\left(1.9+2.95\frac{{W}_{1}}{{W}_{0}}\right)\frac{{W}_{0}}{L}H$$

In Eq. (1), s is the flat-band sensitivity; *d*_31_ is the piezoelectric coefficient; *E* is the Young’s modulus of the piezoelectric material; ω_0_ is the angular resonant frequency of the fundamental mode; *W*_0_ is the width of the fixed end; *W*_1_ is the width of the free ends; *L* is the length of the cantilever from the free to the fixed ends; and *H* is the thickness of the piezoelectric layer. As inferred from Eq. (1), the piezoelectric layer thickness is proportional to the sensitivity, which explains the selection of the 100-µm PVDF film shown in Fig. 1b (the thickest piezoelectric film available from the vendor). Moreover, Eq. (1) indicates that increasing the width/length ratio can enhance the sensitivity for a given resonant frequency (bandwidth) or vice versa. Within the 15 mm-by-15 mm area limit, the preset sensing unit quantity of six allows a maximum width between 3 mm and 4.5 mm. After considering the design redundancy to tolerate possible misalignment during lamination, the width is set to 3.5 mm. Based on the basics of piezoelectric sensing^14^, the length of a single sensing unit is set to 4.2 mm to simultaneously achieve high resonance frequency and sensitivity. With the inertial mass in Fig. 1d, the trapezoid sensing unit has a fundamental resonant frequency of 404.1 Hz, as shown in Fig. 1e. Based on the flat-band sensitivity of each sensing unit in Fig. 1f, the single accelerometer shown in Fig. 1 can achieve a sensitivity of 35.03 pC/g.

### Fabrication of the newly designed accelerometers

**Fig. 2:**
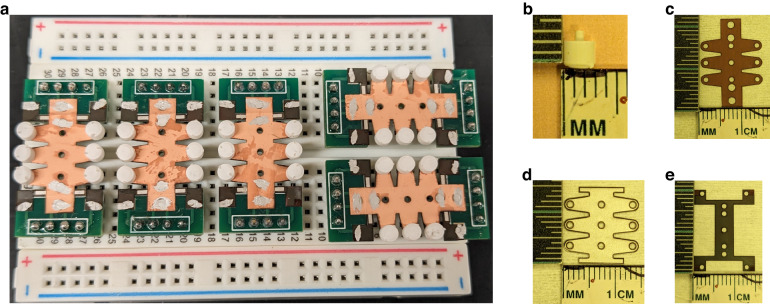
Samples fabricated for the experimental characterization. **a** Five samples of the design in Fig. 2, mounted on a PCB; **b** 3D-printed inertial mass; **c** polyimide surface of the Pi–Cu composite for an accelerometer; **d** PVDF layer for an accelerometer after laser micromachining; **e** polyimide surface of the bottom electrode interconnect layer

Five accelerometers of the design shown in Fig. 1 were manufactured. Figure 2 shows the details of these samples.

As shown in Fig. 2a, we used customized printed circuit boards (PCBs) to interface the samples with the test circuits. The electrical connections were implemented using silver ink, shown as white traces. Figure 2b shows a representative inertial mass used for the five samples in Fig. 2a. In Fig. 2c, the copper-polyimide composite films for the five samples are slightly extended to simplify the electrical connection with the PCB interface. The PVDF films in Fig. 2d are white because the vendor screen-printed conductive silver paint on them. Figure 2e shows the electrical interconnect layer used to access the silver ink on the bottom surface of the PVDF films. Laser milling was conducted at the four corners on the polyimide surface of the interconnect layers to access the copper layer (connected to the bottom surface of the PVDF membrane) from the top surface. Compared with the classic micromachining method of polyimide, our approach provides a simpler and safer alternative. Traditional methods involve hazardous potassium hydroxide solutions at 85 degrees Celsius to wet-etch polyimide^45^ or require specialized reactive ion etching (RIE) equipment for dry etching; thus, they are more perilous, complex, and time-consuming than laser micromachining. In addition, this laser milling process was conducted on the fixed parts of the Pi–Cu composite before the final adhesive lamination with the PVDF layer to minimize the thermal impact on the piezoelectric properties and device performance.

### Experimental characterization of the mechanical resonance characteristics

**Fig. 3:**
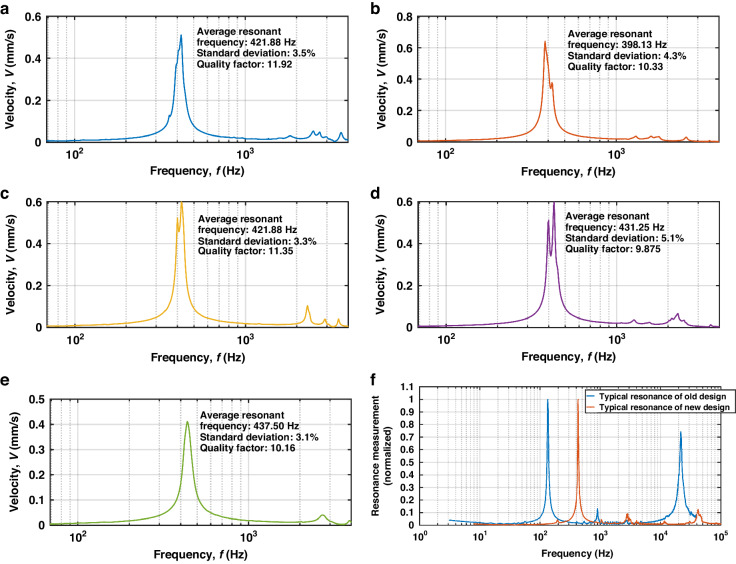
Mechanical resonance measurements using LDV and comparison on a logarithmic scale. **a**–**e** Average resonance spectra for Sample 1, Sample 2, Sample 3, Sample 4, and Sample 5, respectively; **f** Comparison of typical resonance measurements between the new design in Fig. 1 and the old design^29^

We used a Polytec® MSA-500 laser Doppler vibrometer (LDV) to assess the mechanical resonant behaviors of the five samples shown in Fig. 2; our aims were to assess the consistency of their mechanical characteristics, to identify higher-order harmonic modes, and to extract the quality factor for the fundamental mode. The corresponding results are shown in Fig. 3. Please refer to Supplemental Materials C1–C5 for the detailed measurements of each sample.

**Fig. 4:**
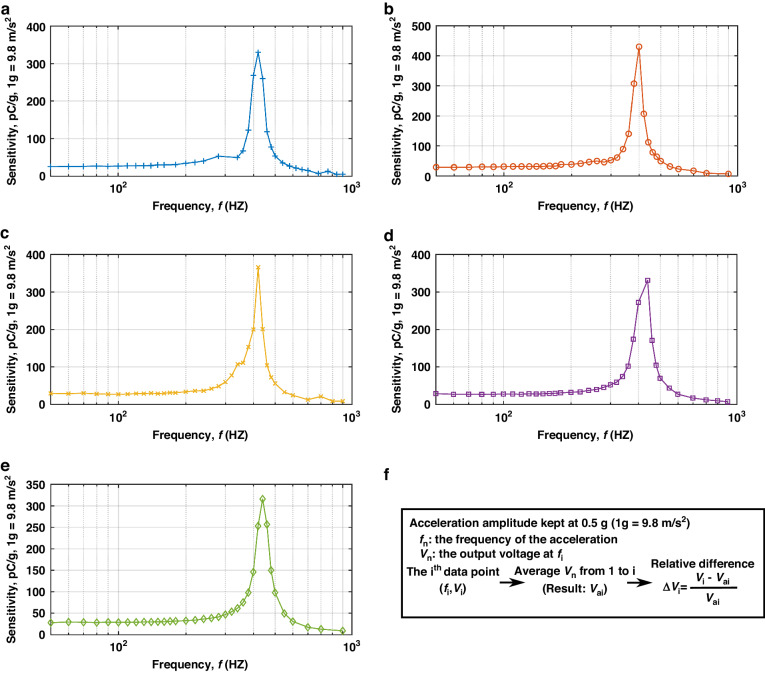
Frequency response to the input accelerations of the five samples for the new design. **a**–**e** Frequency response, logarithmic scale, for Sample 1, Sample 2, Sample 3, Sample 4, and Sample 5, respectively; **f** Method to determine the 5% flat bandwidth

Figure 3a–e provides the statistical data on the mechanical resonance measurements for the five accelerometers. The resonance spectrum of each sample is an average from its six sensing units. The inter-device average fundamental resonant frequency is 422.13 Hz, with a 3.54% standard deviation. A 4.5% relative difference from the simulated resonant frequency in Fig. 1e potentially results from the piezoelectric layer’s stiffening under voltage, as observed in existing studies^46^. The abnormally lower resonance frequency of Sample 2 is likely due to the manufacturing defects such as misalignment or weak PCB adhesion. After removing Sample 2, the standard deviation decreased to ~1.8%, signifying acceptable inter-device consistency in the mechanical resonance. In Figs. 3b, c and 4d, the bifurcated peaks around the average resonant frequency reflect deviations in the single cantilever resonances with standard deviations below 5.1%.

Figure 3f compares the resonant behaviors from the five accelerometers of the new design with those of our old design^29^. The typical resonant behavior of the old design is also available in Supplemental Material A: Mechanical characterization of old samples. Both designs exhibit two higher-order harmonic modes in addition to the fundamental mode. However, the new design shows a significant reduction in the responses at these higher-order modes. For the samples of design in Fig. 1, the maximum amplitude of higher-order harmonic modes, as observed in LDV-based vibrational measurements, is only ~12% of the response of the fundamental mode. In contrast, for the old design^29^, the response of the most significant higher-order harmonic mode is more than 70% of the fundamental mode’s response. Based on the significant difference in Fig. 3f, trapezoid cantilevers can effectively suppress higher-order harmonic modes, validating the geometric design approach employed in this study.

### Experimental results from the frequency response

The mechanical resonance measurements in Fig. 3 reveal a dominant fundamental resonant mode in all five accelerometers, justifying the experimental examination of their sensing capabilities. These five samples were subjected to acceleration-sensing tests on a shaker. Initial tests involved frequency characteristic assessments of sensitivity at a fixed input acceleration of 0.5 g (1 g = 9.8 m/s^2^), with frequencies ranging from 50 to 1000 Hz. The experimentally obtained frequency characteristics are illustrated in Fig. 4. Supplemental Materials C1–C5 provide the detailed measurements of each sample.

In Fig. 4, the voltage readings from the oscilloscope were converted to electrical charges based on the readout circuit design. The sensitivity peaks were observed around the mechanical resonance frequency in Fig. 4a–e. The samples with lower resonance frequencies had higher peak amplitudes. The sensitivity of all five samples was relatively constant within the 0–200 Hz range. The exact bandwidth for each sample was determined using a rolling average method (Fig. 4f). The last frequency where Δ*V*_ai_ falls below 5% is considered the upper limit of the 5% flat band. The last frequency where Δ*V*_ai_ falls below 10% is considered the upper limit of the 10% flat band. For all samples, the upper limit of the 5% band was 160 Hz, while the upper limit of the 10% band was 200 Hz.

### Experimental evaluation of the flat-band sensitivity

**Fig. 5:**
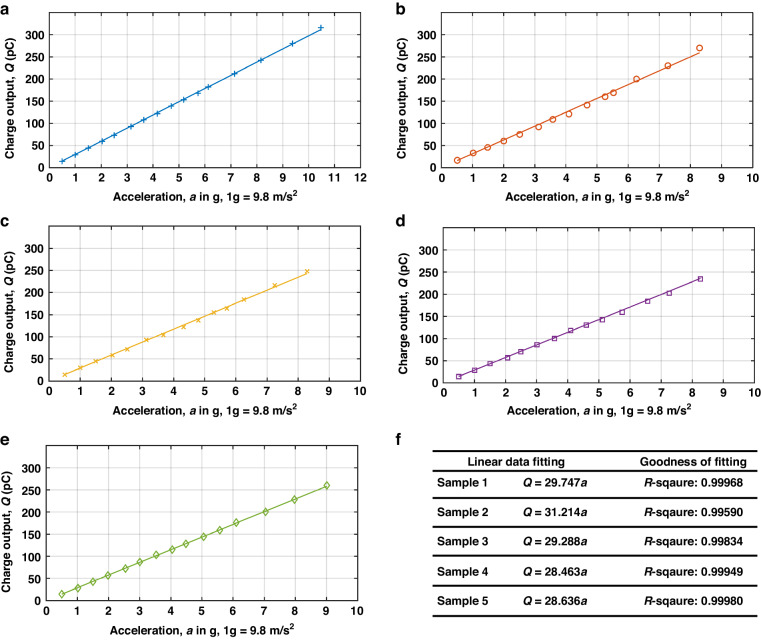
Flat-band sensitivity test for the five samples of the new design. **a**–**e** Response to acceleration from 0 to 10 g (1 g = 9.8 m/s^2^) at 130 Hz for Sample 1, Sample 2, Sample 3, Sample 4, and Sample 5, respectively; **f** Linear fitting of the experimental test results to determine the in-band sensitivity

The experimental determination of the flat-band sensitivity for the five samples in Fig. 2 used the same equipment setup as for the frequency response characterization. The input acceleration frequency is fixed at 130 Hz, while its amplitude varies from 0.5 g to 10 g. The corresponding measurements are plotted in Fig. 5. Supplemental Materials C1–C5 provide the detailed measurements of each sample.

**Fig. 6:**
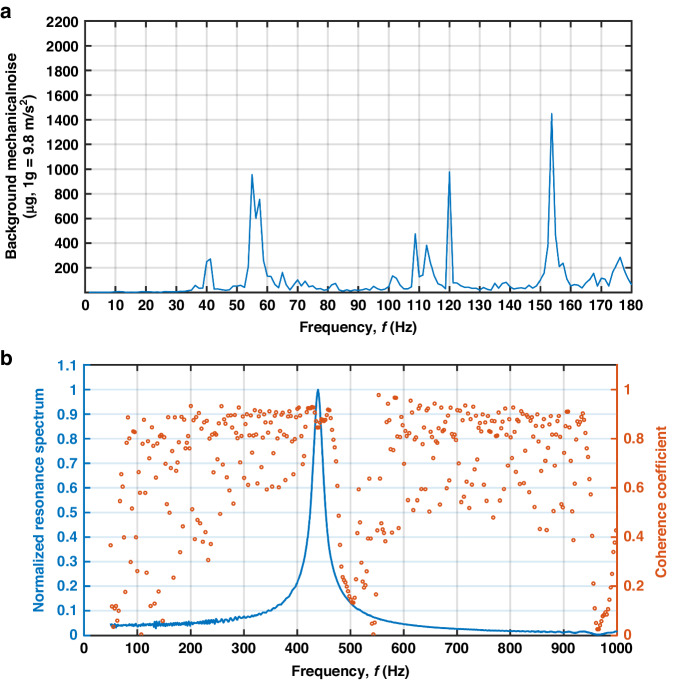
LDV-based noise measurement results. **a** Noise spectrum within the 5% flat band. **b** Representative coherence analysis results for the five accelerometers

In Fig. 5, the voltage readings were converted to electrical charge in a way similar to those shown in Fig. 4. All five accelerometers displayed highly linear in-band responses to input accelerations, with R-square coefficients approaching 1, as shown in Fig. 5f. Sample 2 exhibited a slightly higher flat-band sensitivity, possibly due to its lower fundamental resonance frequency, as shown in Fig. 3b, according to Eq. (1). The average experimentally determined flat-band sensitivity in Fig. 6 was 29.45 pC/g, with a 3.74% standard deviation, reaching 84.1% of the simulated value (35.03 pC/g); thus, using trapezoid cantilevers as sensing units to suppress higher-order harmonic modes could enhance the sensitivity predictability of polymeric piezoelectric MEMS accelerometers.

### Experimental analysis of the noise level

For the noise-level evaluation, first, we focused the LDV on the surface of the inertial mass of the five accelerometers to measure the noise spectrum within the experimentally determined 5% flat band, and the results are shown in Fig. 6.

In Fig. 6a, the noise peaks with amplitudes exceeding 1 mg (1 g = 9.8 m/s^2^) are visible at approximately 60 Hz, 120 Hz, and 160 Hz. Compared to the input acceleration amplitude during sensing tests, these noise peaks are more than a thousand times smaller, signifying minimal disruption to the experimentally determined performance metrics. In Fig. 6b, the coherence coefficients around the peaks observed in Fig. 6a are close to zero, indicating that these peaks are unrelated to the mechanical characteristics but stem from the background environment. Consequently, for the five accelerometers developed in this study, their intrinsic noise properties need to be assessed using the parameters determined in the preceding sections. The classic expressions of the thermomechanical and thermoelectrical noise for piezoelectric accelerometers are as follows^33^:

2 $$\left\{\begin{array}{l}{a}_{m}=\sqrt{\frac{4{k}_{b}T{\omega }_{0}}{Q{m}_{effective}}}\\ {a}_{e}=\sqrt{\frac{4{k}_{b}T\eta C}{\omega {Q}_{c}^{2}}}\end{array}\right.$$

In Eq. (2), *k*_b_ is Boltzmann’s constant; T is the temperature in Kelvin; *ω*_0_ is the angular mechanical resonant frequency; *Q* is the quality factor; *m*_effective_ is close to the inertial mass of the cantilever-based sensing unit; *η* is the dissipation constant of the piezoelectric material (for PVDF: 0.02^47^); C is the total capacitance of the piezoelectric sandwich layer (for the new design in Fig. 3: 174.19 pF); *ω* is the angular frequency of the acceleration; and *Q*_c_ is the charge sensitivity. Based on the power spectrum density (PSD) method, for the design and samples in this study, the two types of noise density can be computed by the following:

3 $$\left\{\begin{array}{l}{a}_{m\_total}={a}_{m}\sqrt{6}\\ {a}_{e\_average}=\sqrt{\frac{\frac{4{k}_{b}T\eta C}{{Q}_{c}^{2}}\,{\mathrm{ln}}\,\frac{{f}_{top}}{{f}_{bottom}}}{{f}_{top}-{f}_{bottom}}}\\ {a}_{total}=\sqrt{{a}_{m}^{2}+{a}_{e}^{2}}\end{array}\right.$$

In Eq. (3), f_top_ is the upper limit of the 5% flat band, determined experimentally at 160 Hz, while f_bottom_ corresponds to the lower cutoff frequency of the readout circuit, set at 1.6 Hz. Among the five accelerometers in Fig. 2, the average thermomechanical noise density is computed as 0.113 µg/$\sqrt{{Hz}}$, with a standard deviation of 4.2%. The average thermoelectrical noise density is computed as 1.39 µg/$\sqrt{{Hz}}$, with a standard deviation of 3.7%. The new design exhibits an average in-band noise density of 1.40 µg/$\sqrt{{Hz}}$, with a standard deviation of 3.63%, featuring a thermoelectrical noise that is ten times greater; these results are in agreement with findings from existing studies^16,33^ on the dominance of the thermoelectrical noise in piezoelectric accelerometers. Furthermore, the noise from the readout circuit, utilizing the AD8608 operational amplifier with a noise density of 8 nV/$\sqrt{{Hz}}$^48^, corresponds to a 0.27 µg/$\sqrt{{Hz}}$ distortion in the in-band noise density, as per the experimental results.

## Discussion

For the five accelerometers of the design in Fig. 1, their experimentally obtained performance has an acceptable alignment with the simulations. To evaluate whether these performance metrics can fulfill the objectives of this study, Table 1 shows a comparison of the experimental performance metrics from the five accelerometers in Fig. 2 with those of existing piezoelectric accelerometers, including other polymeric accelerometers and PZT-based accelerometers.

**Table 1:** Comparison of the performance metrics between the samples of the new designs from this study and those from other existing studies

Work	Sensitivity (pC/g)	Resonant frequency (Hz)	Bandwidth (Hz)	Noise density (µg/ $\sqrt{{Hz}}$)	Sensing unit area (mm^2^)	Piezoelectric material
This work	29.45 ( ± 3.74%)	422.13	160 (5%)	1.4 ( ± 0.27, in-band average)	88.2 (6 units)	PVDF
			200 (10%)	0.7271 @ 20 Hz		
Ge et al.^29^	21.82 ( ± 4.08%)	128.95	58.6 (5%)	2.75 ( ± 0.37, in-band average)	90.0 (6 units)	PVDF
				0.975 @ 20 HZ		
Hewa-Kasakarage et al.^17^	5.10	364.30	N/A	1.74 @ 20 Hz	0.19	PZT
	50.00	67.00	N/A	1.3 @50 Hz	0.44	
Gong et al.^15^	25.60	769.01	150 (5%)	N/A	10.5	PZT
	25.50	572.25	110 (5%)		6.00	
	41.40	573.59	110 (5%)		10.5	
	23.90	723.25	140 (5%)		7.725	
Gong et al.^16^	22.74	867.4	200 (5%)	5.6 @ 20 Hz	9.00	PZT
Wang et al.^28^	28.95 ( ± 20.67%)	87.5	15	N/A	200	Cellulose mixed with ZnO
Gong et al.^27^	134.59 (around resonant frequency)	~150	N/A	N/A	150	PVDF

As shown in Table 1, the PVDF-based polymeric piezoelectric accelerometers presented in this study outperform the PZT-based accelerometers despite their inferior material properties. Our polymeric accelerometers have a higher flat-band sensitivity than six of seven PZT-based accelerometers and a broader bandwidth than four of the six. Moreover, our accelerometers exhibit significantly lower noise levels, possibly due to the substantial dielectric constant difference between PVDF (~13.5) and PZT (~1300). As for the comparison with existing polymeric counterparts, Table 1 shows that our PVDF-based polymeric piezoelectric accelerometers are among the top performers in terms of sensitivity, flat bandwidth, noise levels, and performance consistency. This performance demonstrates the high potential of the PVDF-based polymeric piezoelectric accelerometers developed in this study as vibration sensors for various wearable microsystems, such as vocal vibration amplifiers/assistants. To implement these practical applications, critical research in the next phase is developing a proper packaging method. As noted by Gong et al.^16^, piezoelectric accelerometers do not need vacuum sealing, which is the basis of simple packaging processes based on 3D-printed polymer components. Recently, Liu et al. experimentally demonstrated the feasibility of using a 3D-printed package to monolithically integrate a MEMS accelerometer and a force sensor^49^. Aspar et al. experimentally demonstrated that stereolithography (STL) 3D printing techniques could be used to develop polymeric MEMS packages^50^ that could hold sensors and flexible/rigid PCBs with dimensions suitable for polymeric wearable smart systems^9,39,51^.

Table 1 also explicitly shows the limitations of polymeric piezoelectric accelerometers based on the classic piezoelectric sensing principle. Although the samples of the design in Fig. 1 are experimentally validated to have sensitivity, bandwidth, and noise levels that are comparable to those of their PZT-based counterparts, their size is still significantly larger. This limited degree of miniaturization can be considered inevitable for polymeric piezoelectric accelerometers based on the classic piezoelectric sensing principle since the output electrical charge has the following relationship with the device sensing area and material properties^14^:

4 $${Q}_{piezo}\propto dEH\int\int dA$$

In Eq. (4), *d* is the piezoelectric coefficient, *E* is the Young’s modulus of the piezoelectric material, H is the thickness of the piezoelectric material, and the integral is the area integral of the sensing unit. Since polymeric piezoelectric materials cannot compete with their inorganic counterparts for material properties, as per Eq. (4), to achieve comparable performance, the dimensions of polymeric piezoelectric accelerometers inevitably have to be larger. To further miniaturize polymeric piezoelectric accelerometers while maintaining competitive performance, one possible solution is to use organic piezoelectric field-effect transistors (PFETs) as the fundamental sensing units. A PFET uses mechanical stress to control the formation of the carrier channel between the drain and the source. Previous studies have used PVDF-based PFETs as tactile sensors^52^. The use of PVDF-based PFETs for polymer accelerometers will require dedicated study into the materials for electronics, PFET transistor structures, and interface design.

## Methods

Our fabrication process is the same as the one employed in our previous design^29^. PVDF sheets and polyimide-copper composites undergo laser micromachining, while the inertial mass is created via stereolithography 3D printing. Separately manufactured components are assembled using adhesive lamination, aided by a 3D-printed reference base for alignment. Notably, this microfabrication approach relies on direct polymeric micromachining techniques, omitting the repetitive cycles of material deposition, masking lithography, and anisotropic etching required in traditional micromachining flows. However, this current microfabrication flow is quite unstandardized and involves significant manual labor. To address this issue and increase the scalability of the fabrication process, roll-to-roll lamination flows with setups and methods used in printed circuit board manufacturing could be potentially combined. Studies have experimentally demonstrated that roll-to-roll lamination can be used to mass-produce polymeric piezoelectric transducers^5^ and photodetectors^4^. Laser micromachining has been a standard method used in the production pipelines of PCBs for years. In addition to patterns with dimensions ranging from micrometers to millimeters, the corresponding standardized manufacturing flow also involves automated, accurate alignment between the layers and the dicing process.

Our accelerometer experiment setup resembles our previous work^29^ and employs a Polytec^®^ MSA-500 Laser Doppler Vibrometer (LDV) for mechanical resonance/vibration measurements, with scanning points on the sample surfaces. Electrical actuation is achieved through amplified voltage signals from the LDV, which also monitors input accelerations during bandwidth and sensitivity evaluations. Vibration excitation is provided by a Dataphysics^®^ V4 shaker, while the electrical readout circuit comprises two Analog Device^®^ CN0350 boards and a Siglent^®^ SDS 1202X-E oscilloscope.

More details regarding the microfabrication procedure and experimental setup can be found in our previous publication^29^.

## Conclusions

In this study, a way to overcome material limitations was experimentally provided, and PVDF-based polymeric piezoelectric accelerometers that have high sensitivity, broad bandwidth, and low noise levels were developed. Compared to other traditional polymeric piezoelectric accelerometers, our new PVDF-based polymeric piezoelectric accelerometer design used an array of smaller sensing units to obtain a high resonance frequency while maintaining a similar total sensing area. Trapezoid cantilevers whose dimensional parameters were numerically optimized were used to enhance the fundamental resonant mode and maintain an actual sensitivity close to its theoretical ceiling. Experimental tests on the five prototypes yielded remarkable results: an average flat-band sensitivity of 29.45 pC/g, a 5% flat band of 160 Hz, a 10% flat band of 200 Hz, and an in-band noise density of 1.4 µg/$\sqrt{{Hz}}$. These achievements position our accelerometers among the best polymeric piezoelectric accelerometers, and our accelerometers even outperform several PZT-based counterparts. Based on the experimentally confirmed metrics, the PVDF-based polymeric piezoelectric accelerometers in this study could be considered viable candidates for vibration sensors in wearable organic microsystems and electronics.

### Supplementary information

Supplement material A Mechanical characterization of old samples

Supplement material B Math model derivation

Supplement material C1 Measurement result for Sample 1

Supplement material C2 Measurement result for Sample 2

Supplement material C3 Measurement result for Sample 3

Supplement material C4 Measurement result for Sample 4

Supplement material C5 Measurement result for Sample 5

## References

[1] Ismar E, Bahadir SK, Kalaoglu F, Koncar V (2020). Futuristic clothes: electronic textiles and wearable technologies. Glob. Chall..

[2] Lu CY (2016). Progress in flexible organic thin-film transistors and integrated circuits. Sci. Bull..

[3] Xie, Y. F. et al. Organic transistor-based integrated circuits for future smart life. *SmartMat* **37**, e1261 (2024).

[4] Xia YX, Aguirre LE, Xu XF, Inganäs O (2020). All-polymer high-performance photodetector through lamination. Adv. Electron. Mater..

[5] Schmidt GC (2021). Paper-embedded roll-to-roll mass printed piezoelectric transducers. Adv. Mater..

[6] Paula KT (2018). Femtosecond laser micromachining of polylactic acid/graphene composites for designing interdigitated microelectrodes for sensor applications. Opt. Laser Technol..

[7] Fisher C, Skolrood LN, Li K, Joshi PC, Aytug T (2023). Aerosol-jet printed sensors for environmental, safety, and health monitoring: a review. Adv. Mater. Technol..

[8] Karipoth P (2024). Aerosol jet printing of strain sensors for soft robotics. Adv. Eng. Mater..

[9] Osman A, Lu J (2023). 3D printing of polymer composites to fabricate wearable sensors: a comprehensive review. Mater. Sci. Eng. R Rep..

[10] Cichosz S, Masek A, Zaborski M (2018). Polymer-based sensors: a review. Polym. Test..

[11] Tao XL, Liao SL, Wang YP (2021). Polymer-assisted fully recyclable flexible sensors. EcoMat.

[12] Tadigadapa S, Mateti K (2009). Piezoelectric MEMS sensors: state-of-the-art and perspectives. Meas. Sci. Technol..

[13] Algamili AS (2021). A review of actuation and sensing mechanisms in MEMS-based sensor devices. Nanoscale Res. Lett..

[14] Polcawich, R. G. & Pulskamp, J. S. In *MEMS Materials and Processes Handbook* (eds Reza, G. & Pinyen L.) 273–353 (Springer US, 2011).

[15] Gong, X., Chen, C.-T., Wu, W.-J. & Liao, W.-H. In *Sensors and Smart Structures Technologies for Civil, Mechanical, and Aerospace Systems 2019, March 4, 2019–March 7, 2019* (OZ Optics, Ltd.; Polytec, Inc.; The Society of Photo-Optical Instrumentation Engineers (SPIE, 2019).

[16] Gong X, Kuo Y-C, Zhou G, Wu W-J, Liao W-H (2023). An aerosol deposition based MEMS piezoelectric accelerometer for low noise measurement. Microsyst. Nanoeng..

[17] Hewa-Kasakarage NN, Kim D, Kuntzman ML, Hall NA (2013). Micromachined piezoelectric accelerometers via epitaxial silicon cantilevers and bulk silicon proof masses. J. Microelectromech. Syst..

[18] Polla DL, Francis LF (1996). Ferroelectric thin films in microelectromechanical systems applications. MRS Bull..

[19] Liu Y (2021). A novel tri-axial piezoelectric MEMS accelerometer with folded beams. Sensors.

[20] Corman, R., Nedelcu, O. & Dobrescu, D. In *2016**International Semiconductor Conference (CAS)* 85–88 (IEEE, 2016).

[21] Gesing AL, Alves FDP, Paul S, Cordioli JA (2018). On the design of a MEMS piezoelectric accelerometer coupled to the middle ear as an implantable sensor for hearing devices. Sci. Rep..

[22] Neves Masson Z, Gesing AL, de Lorenzo M, Paul S, Cordioli JA (2018). A new approach for an FE-model optimization of a MEMS piezoelectric accelerometer for implantable hearing devices. J. Acoust. Soc. Am..

[23] Li W (2017). Sensitivity-enhanced wearable active voiceprint sensor based on cellular polypropylene piezoelectret. ACS Appl. Mater. Interfaces.

[24] Wang HS (2021). Biomimetic and flexible piezoelectric mobile acoustic sensors with multiresonant ultrathin structures for machine learning biometrics. Sci. Adv..

[25] Fitch JL, Holbrook A (1970). Modal vocal fundamental frequency of young adults. Arch. Otolaryngol..

[26] Ramadan KS, Sameoto D, Evoy S (2014). A review of piezoelectric polymers as functional materials for electromechanical transducers. Smart Mater. Struct..

[27] Gong Y, Zhao H, Huang Y, Jin X (2021). Design and experimental study of acceleration sensor based on PVDF piezoelectric film. J. Comput. Methods Sci. Eng..

[28] Wang YH (2018). A paper-based piezoelectric accelerometer. Micromachines.

[29] Ge C, Cretu E (2023). A polymeric piezoelectric MEMS accelerometer with high sensitivity, low noise density, and an innovative manufacturing approach. Microsyst. Nanoeng..

[30] Pan X (2016). A self-powered vibration sensor based on electrospun poly(vinylidene fluoride) nanofibres with enhanced piezoelectric response. Smart Mater. Struct..

[31] Jin L (2018). Polarization-free high-crystallization β-PVDF piezoelectric nanogenerator toward self-powered 3D acceleration sensor. Nano Energy.

[32] Wei X (2021). A piezoelectric power generator based on axisymmetrically distributed PVDF array for two-dimension vibration energy harvesting and direction sensing. Sustain. Energy Technol. Assess..

[33] Levinzon FA (2004). Fundamental noise limit of piezoelectric accelerometer. IEEE Sens. J..

[34] Mohammadpourfazeli S (2023). Future prospects and recent developments of polyvinylidene fluoride (PVDF) piezoelectric polymer; fabrication methods, structure, and electro-mechanical properties. RSC Adv..

[35] Karan SK (2016). An approach to design highly durable piezoelectric nanogenerator based on self-poled PVDF/AlO-rGO flexible nanocomposite with high power density and energy conversion efficiency. Adv. Energy Mater..

[36] Rana MM (2020). Porosity modulated high-performance piezoelectric nanogenerator based on organic/inorganic nanomaterials for self-powered structural health monitoring. ACS Appl. Mater. Interfaces.

[37] Park S-J, Kim J, Chu M, Khine M (2018). Flexible piezoresistive pressure sensor using wrinkled carbon nanotube thin films for human physiological signals. Adv. Mater. Technol..

[38] Wei H (2021). Polypyrrole/reduced graphene aerogel film for wearable piezoresisitic sensors with high sensing performances. Adv. Compos. Hybrid. Mater..

[39] De Fazio R, Stabile M, De Vittorio M, Velázquez R, Visconti P (2021). An overview of wearable piezoresistive and inertial sensors for respiration rate monitoring. Electronics.

[40] Karihaloo BL, Niordson FI (1973). Optimum design of vibrating cantilevers. J. Optim. Theory Appl..

[41] Goel RP (1976). Transverse vibrations of tapered beams. J. Sound Vib..

[42] Wang CY (2013). Vibration of a tapered cantilever of constant thickness and linearly tapered width. Arch. Appl. Mech..

[43] Sahin O (2004). High-resolution imaging of elastic properties using harmonic cantilevers. Sens. Actuators A: Phys..

[44] Raman A, Melcher J, Tung R (2008). Cantilever dynamics in atomic force microscopy. Nano Today.

[45] Kreuz, John A. & Christopher M. Hawkins. High speed etching of polyimide film. U.S. Patent No. 4,426,253 (1984).

[46] Waisman H, Abramovich H (2002). Active stiffening of laminated composite beams using piezoelectric actuators. Composite Struct..

[47] Moffett, M. B., Powers, J. M. & NAVAL UNDERWATER SYSTEMS CENTER NEW LONDON CT. Dielectric Properties of Piezoelectric Polyvinylidene Fluoride (PVDF). NUSC Technical Memorandum 841072, (1984).

[48] Precision, Low Noise, CMOS, Rail-to-Rail, Input/Output Operational Amplifiers, AD8605/AD8606/AD8608, Rev. O, Analog Devices, 2017. Available: https://www.analog.com/media/en/technical-documentation/data-sheets/ad8605_8606_8608.pdf.

[49] Liu, G. D. et al. In *36th IEEE International Conference on Micro Electro Mechanical Systems (MEMS)* 598–601 (IEEE, 2023).

[50] Aspar, G. et al. In *IEEE 67th Electronic Components and Technology Conference (ECTC)* 1071–1079 (IEEE, 2017).

[51] Yang TT, Xie D, Li ZH, Zhu HW (2017). Recent advances in wearable tactile sensors: Materials, sensing mechanisms, and device performance. Mater. Sci. Eng. R.-Rep..

[52] Wang J (2020). Energy-efficient, fully flexible, high-performance tactile sensor based on piezotronic effect: piezoelectric signal amplified with organic field-effect transistors. Nano Energy.

