# Supplementary material for: Polymeric piezoelectric accelerometers with high sensitivity, broad bandwidth, and low noise density for organic electronics and wearable microsystems
Source: Microsyst Nanoeng. 2024 May 15;10:61. doi: 10.1038/s41378-024-00704-6 (PMC11093978; doi:10.1038/s41378-024-00704-6)
Supplement: Supplementary file 1 — Supplement material A Mechanical characterization of old samples [file 41378_2024_704_MOESM1_ESM.docx]

**Supplement material A: mechanical characterization of old samples**

For accelerometers, the tradeoff relationship between sensitivity and resonant frequency (or flat bandwidth) only stands when the sensitivity of the MEMS accelerometer is close to its theoretical ceiling. Suppose the experimentally determined sensitivity of a polymeric piezoelectric MEMS accelerometer is far below this ceiling. Therefore, it becomes vital for the research in this paper to initiate by examining whether the polymeric piezoelectric MEMS accelerometers in our prior publication^1^ have any sensitivity-related underperformance and determining potential mechanisms.

We have employed laser Doppler vibrometry (LDV) to conduct an in-depth characterization of the mechanical vibration behavior of our previous PVDF-based polymeric piezoelectric MEMS accelerometers^1^. Our previous design featured six identical cantilever structures electrically connected in parallel as sensing units within a single device, using their combined piezoelectric response as output. These accelerometers exhibited an average fundamental resonant frequency of 128.95 Hz and an experimentally determined average sensitivity of 21.82 pC/g, with a 5% flat bandwidth of 58.5 Hz (1g=9.8m/s^2^). Figure S1 illustrates the corresponding results.


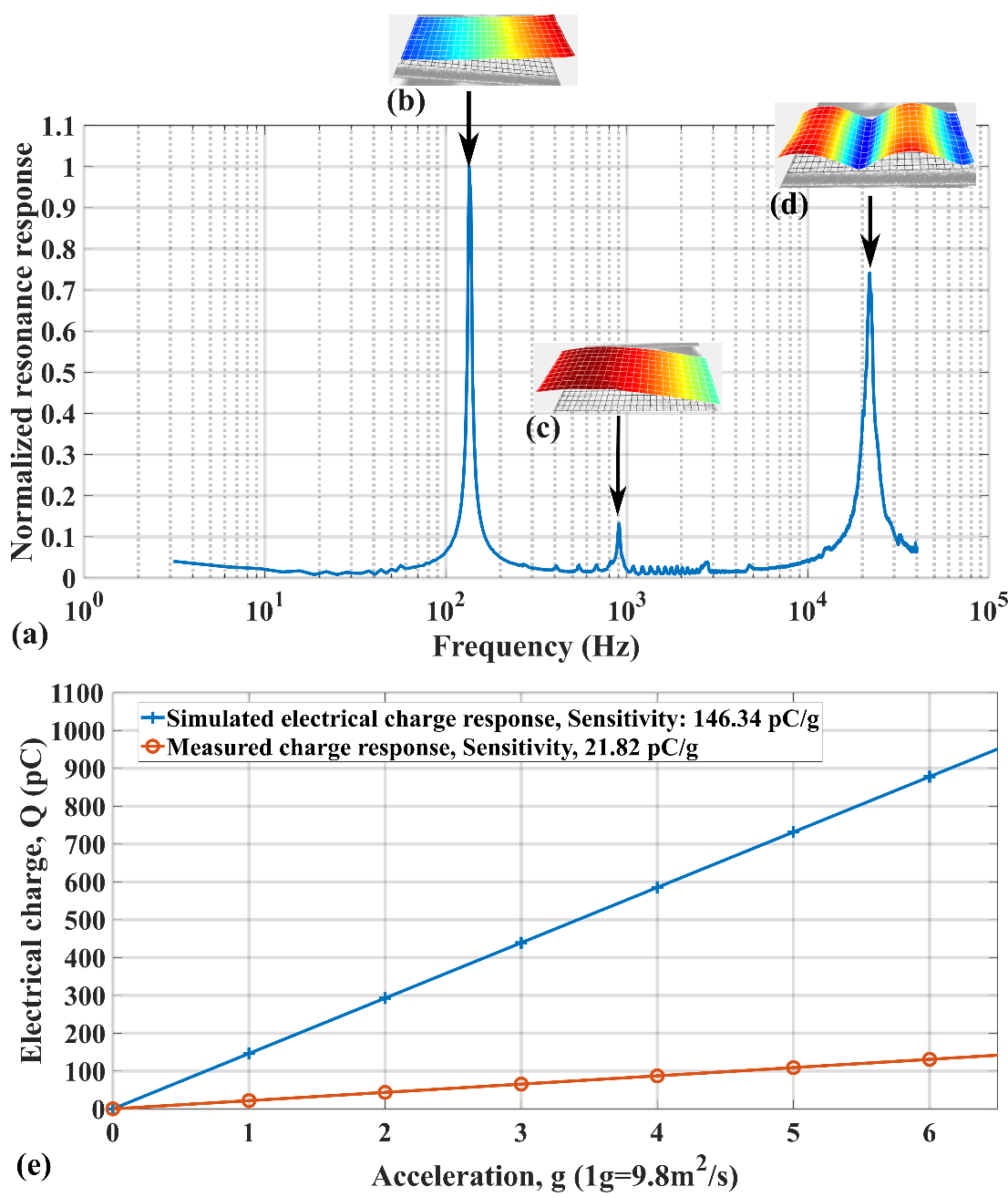


Figure S1 Resonant behavior measurement results and comparison between theoretical and measured sensitivity for the authors' previous polymeric piezoelectric MEMS accelerometer designs^1^. (a) Frequency spectrum of the resonant behaviors, logarithmic scale, 0 to 40 kHz; (b) to (d): Representative modal shapes visible in the LDV measurements on the sensing cantilever surface; (b): The fundamental mode; (c): The third mode; (d): The 15^th^ mode; (e): Comparison between the measured and simulated flat-band sensitivity.

The LDV measurements yielded resonant peaks (Figure S1a) and corresponding modal shapes (Figures S1b to 1d). In Figure S1a, we normalized the resonant peaks for more convenient analysis, with their harmonic orders identified by matching measured modal shapes with COMSOL Multiphysics 6.1 simulation results. While the fundamental mode (1st mode) exhibited the most significant response, the 3rd and 15th modes also displayed observable responses in Figure 1a. The response of the 3rd mode was an order of magnitude smaller than that of the fundamental mode, whereas the response of the 15th mode reached over 70% of the fundamental mode's response.

The unsuppressed 15^th^ mode in the LDV measurements (Figure S1a) could potentially impact the sensitivity of the cantilever-based polymeric piezoelectric MEMS accelerometers. Fundamental theories regarding cantilever vibrations^2^ and piezoelectric charge generation^3^ suggest that the responses of all unsuppressed resonant modes contribute to the final output of a cantilever-based piezoelectric MEMS accelerometer in a weighted manner. For each prominent resonant mode, the quantity of piezoelectric charges generated is influenced by the stress amplitude and its distribution across the surface. Stress amplitude depends on the resonant frequency and input acceleration conditions, while stress distribution follows the modal shape^2^. In the case of the fundamental mode, as shown in Figure S1b, stress distribution remains consistently compressive or tensile along the surface, accumulating electrical charges at the top and bottom surfaces of the piezoelectric layer. Conversely, for the 15^th^ mode, as depicted in Figure S1d, the alternating stress component results in stress across the surface fluctuating between compressive and tensile, mostly canceling out and generating fewer electrical charges. Consequently, the total electrical output of our prior design^1^ primarily reflects the weighted response of the fundamental mode, lower than the theoretical ceilings. The impact of the unsuppressed higher harmonic mode is evident in Figure S1e. Despite rivaling some PZT-based accelerometers, our old devices^1^ exhibited only 15% of the simulated sensitivity.

The experimental discovery in Figure 1 is a cornerstone for the feasibility of the study in this paper. It indicates that effectively suppressing higher-order harmonic modes in cantilever-based accelerometers could enable us to implement a new design with a higher resonant frequency and broader bandwidth while preserving or surpassing the experimentally determined sensitivity from the prior design^1^.

**Reference**

1 Ge, C. & Cretu, E. A polymeric piezoelectric MEMS accelerometer with high sensitivity, low noise density, and an innovative manufacturing approach. *Microsystems & Nanoengineering* **9**, 151 (2023). <https://doi.org:10.1038/s41378-023-00628-7>

2 Volterra, E., Zachmanoglou, E. C. & Kolsky, H.

3 Polcawich, R. G. & Pulskamp, J. S. in *MEMS Materials and Processes Handbook* (eds Reza Ghodssi & Pinyen Lin) 273-353 (Springer US, 2011).
