# Supplementary material for: Polymeric piezoelectric accelerometers with high sensitivity, broad bandwidth, and low noise density for organic electronics and wearable microsystems
Source: Microsyst Nanoeng. 2024 May 15;10:61. doi: 10.1038/s41378-024-00704-6 (PMC11093978; doi:10.1038/s41378-024-00704-6)
Supplement: Supplementary file 2 — Supplement material B Math model derivation [file 41378_2024_704_MOESM2_ESM.docx]

**Supplement material B: Math model derivation**

Assuming a piezoelectric MEMS accelerometer with the fundamental mode as the only prominent mode, the amplitude A(ω_0_,ω) and deflection D(x) for its mechanical response to external acceleration have the following general forms^1^:

$$\left\{\begin{matrix} A(\omega_{0},\omega)=\frac{a_{input}}{\sqrt{\left(\omega_{0}^{2}-\omega^{2}\right)^{2}+\left(\frac{b\omega}{m_{0}}\right)^{2}}} \\ D(x)=C_{1}\cos(k_{1}x)+C_{2}\cosh(k_{1}x)+C_{3}\sin(k_{1}x)+C_{4}\sinh(k_{1}x) \end{matrix}\right.$$

In Eq, a_input_ is the input acceleration amplitude; m_0_ is the effective mass for the fundamental resonant mode; b is the viscous damping coefficient; ω_0_ is the angular resonant frequency of the fundamental mode; ω is the angular frequency of the input acceleration; x is the distance of a location on the cantilever to the fixed end; C_1_ to C_4_ are constants derivable with boundary conditions; k_1_ is a constant related to the material properties and structure dimensions. Assuming the cantilever-based MEMS accelerometer has a length of L and a fully static initial condition as below:

$$\left\{\begin{matrix} D(0)=0 \\ \left.\frac{\partial D}{dx}\right|_{x=0}=0 \\ \left.\frac{\partial^{2}D}{dx^{2}}\right|_{x=L}=0 \\ \left.\frac{\partial^{3}D}{dx^{3}}\right|_{x=L}=0 \end{matrix}\right.$$

Based on Eq, D(x) in Eq can be rewritten as:

$$D(x)=C\left\{\left[\cos(k_{1}x)-\cosh(k_{1}x)\right]+\left[\frac{-\cos(k_{1}L)-\cosh(k_{1}L)}{\sin(k_{1}L)+\sinh(k_{1}L)}\right]\left[\sin(k_{1}x)-\sinh(k_{1}x)\right]\right\}$$

The following condition also stands:

$$\cos(k_{1}L)\cosh(k_{1}L)=-1$$

In classic structural mechanics, Eq is the frequency equation for a cantilever^1^. The first non-zero solution of Eq is 1.875 (in radian units). Replacing the k_1_L in Eq with this value, we get the quantitative relationship for the forced vibration response of a cantilever-based piezoelectric MEMS accelerometer:

$$R(\omega,x)\propto\frac{a_{input}\left\{\left[\cos(\frac{1.875}{L}x)-\cosh(\frac{1.875}{L}x)\right]-0.734\left[\sin(\frac{1.875}{L}x)-\sinh(\frac{1.875}{L}x)\right]\right\}}{\sqrt{\left(\omega_{0}^{2}-\omega^{2}\right)^{2}+\left(\frac{b\omega}{m_{0}}\right)^{2}}}$$

The stress within a surface can be expressed using Eq and the beam bending theories^2^ as:

$$\left\{\begin{matrix} M=EI\frac{\partial^{2}R(\omega,x)}{\partial x^{2}} \\ \sigma=\frac{M}{I}z\propto\frac{a_{input}E\left(\frac{1.875}{L}\right)^{2}z\left\{\left[\cos(\frac{1.875}{L}x)+\cosh(\frac{1.875}{L}x)\right]+1.3623\left[\sin(\frac{1.875}{L}x)+\sinh(\frac{1.875}{L}x)\right]\right\}}{\sqrt{\left(\omega_{0}^{2}-\omega^{2}\right)^{2}+\left(\frac{b\omega}{m_{0}}\right)^{2}}} \end{matrix}\right.$$

In Eq, M is the bending moment; I is the moment of inertia of the cantilever-based sensing structure; E is the Young's modulus of the cantilever structural material; z is the distance of a surface to the neutral axis of the cantilever. The integral to get the piezoelectric charge can be written as^3^:

$$Q_{piezo}=\iint d_{31}\left(\sigma_{top}-\sigma_{bottom}\right)dA$$

In Eq, d_31_ is the piezoelectric coefficient that transfers the elongating stress during a bending into the piezoelectric charge. For a horizontally tapered cantilever, assume a tilting angle of θ, at the location that is away from the fixed end by x, the corresponding width is:

$$W(x)=W_{0}-2\tan(\theta)x$$

Correspondingly, the variation in area is:

$$dA=(W_{0}-2\tan(\theta)x)dx$$

Where W_0_ is the width at the fixed end of the tapered cantilever. Bringing Eq and Eq into Eq, we get:

$$\left\{\begin{matrix} Q_{piezo}\propto\frac{a_{input}E\left(\frac{1.875}{L}\right)^{2}Hd_{31}}{\sqrt{\left(\omega_{0}^{2}-\omega^{2}\right)^{2}+\left(\frac{b\omega}{m_{0}}\right)^{2}}}A \\ A=\int_{0}^{L} (W_{0}-2\tan(\theta)x)\left[\cos(\frac{1.875}{L}x)+\cosh(\frac{1.875}{L}x)\right]+1.3623\left[\sin(\frac{1.875}{L}x)+\sinh(\frac{1.875}{L}x)\right]dx \end{matrix}\right.$$

In Eq, H is the piezoelectric material layer thickness. The definite integral in Eq can be separated and computed as the following:

$$\left\{\begin{matrix} \int_{0}^{L} W_{0}\left[\cos(\frac{1.875}{L}x)+\cosh(\frac{1.875}{L}x)\right]+1.3623\left[\sin(\frac{1.875}{L}x)+\sinh(\frac{1.875}{L}x)\right]dx=4.85LW_{0} \\ \int_{0}^{L} 2\tan(\theta)x\left[\cos(\frac{1.875}{L}x)+\cosh(\frac{1.875}{L}x)\right]+1.3623\left[\sin(\frac{1.875}{L}x)+\sinh(\frac{1.875}{L}x)\right]dx=5.90\tan(\theta)L^{2} \end{matrix}\right.$$

Bringing Eq into Eq, there is:

$$Q_{piezo}\propto\frac{a_{input}}{\sqrt{\left(\omega_{0}^{2}-\omega^{2}\right)^{2}+\left(\frac{b\omega}{m_{0}}\right)^{2}}}d_{31}EH(\frac{4.85W_{0}}{L}-5.9\tan(\theta))$$

Eq can be rewritten using the width of the free-end, W_1_, as below:

$$\left\{\begin{matrix} \tan(\theta)=\frac{(W_{0}-W_{1})}{2L} \\ \begin{matrix} k=\frac{W_{1}}{W_{0}}, & 0\leq k\leq1 \end{matrix} \\ Q_{piezo}\propto\frac{a_{input}}{\sqrt{\left(\omega_{0}^{2}-\omega^{2}\right)^{2}+\left(\frac{b\omega}{m_{0}}\right)^{2}}}d_{31}EH(1.9+2.95k)(\frac{W_{0}}{L}) \end{matrix}\right.$$

The classic theories of MEMS accelerometers^4^ define the flat band as a frequency band where the variation in the electrical output is below 5% or 10%, with the corresponding band called the 5% band or 10% band. Since these flat bands are generally considered to start from the DC signal(ω=0)^4^, we get the following approximation:

$$s=\frac{Q_{piezo}}{a_{input}}\propto d_{31}E\frac{1}{\omega_{0}^{2}}(1.9+2.95k)\frac{W_{0}}{L}H$$

Eq is the same as the equation presented in the paper.

**COMSOL Multiphysics simulation setup to validate the math model**

**Structure 3D design:**


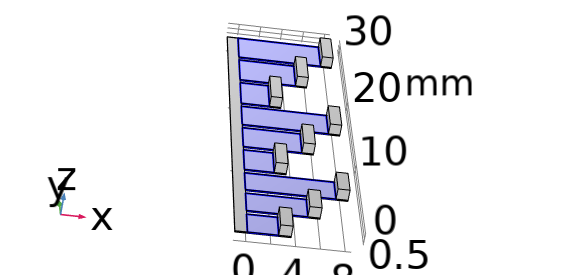


Figure S1: 3D design of the structures for FEA

**Depiction:**

The design consists of 9 rectangular cantilevers. At the free end of a cantilever, there is an inertial mass.

**Planar dimensions:**

The planar dimensions of the nine cantilevers (Blue regions in the Figure S1):

| **Cantilevers** | 1 | 2 | 3 | 4 | 5 | 6 | 7 | 8 | 9 |
| --- | --- | --- | --- | --- | --- | --- | --- | --- | --- |
| **Length (mm)** | 2.5 | 5 | 7.5 | 2.5 | 5 | 7.5 | 2.5 | 5 | 7.5 |
| **Width (mm)** | 2.5 | 2.5 | 2.5 | 3 | 3 | 3 | 3.5 | 3.5 | 3.5 |

As for the inertial mass, the length is the same as the width of the cantilevers, while the width is fixed at 1mm. The height is fixed at 1mm.

**Materials:**

The properties of all materials are from the library of COMSOL Multiphysics 6.1.

Cantilever:

The cantilevers have three layers. From the bottom to the top (Z-axis direction), the material layers are:

- PVDF: Thickness: H (100 µm or 50 µm)
- Polyimide: Thickness: 12 µm
- Copper: Thickness: 9 µm

Inertial mass:

- Polyimide.

**Physics selection:**

AC/DC -> Electromagnetics and Mechanics -> Piezoelectricity, Solid

**Physics setup:**

**Solid Mechanics:**

**Fixed constraints:**

One fixed constraint is applied to the blue regions in Figure S2.


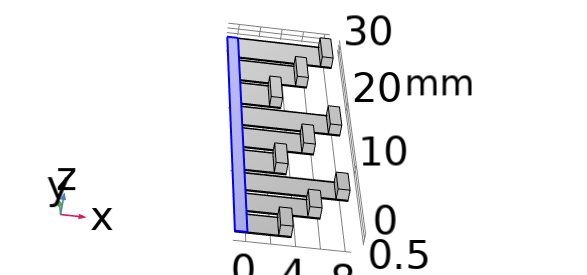


Figure S2: Method to apply the fixed constraint

**Linear Acceleration Frame:**

An acceleration of G is applied to the entire structure. G ranges from 0 to 5g (1g = 9.8m/s^2^). It is only used for the parametric sweep in the stationary study to determine the sensitivity.

**Piezoelectric materials:**

Only the PVDF components are selected.

**Electrostatics:**

Only the PVDF components are selected for the Charge Conservation and Charge Conservation, Piezoelectric.

**Studies:**

Two sets of simulations are conducted, one for a PVDF thickness of 50µm and the other for 100 µm. Each set includes the following studies:

- Eigenfrequency: Physics controlled.
- Stationary: Physics controlled, Parametric sweep: G from 0 to 5g, interval: 1g.

**Results and post-simulation processing:**

For the results of the stationary parametric sweep, a surface integration has been conducted on the bottom and top surfaces of the PVDF layers for each cantilever:

- Selected expression: Solid mechanics -> Piezoelectric -> Piezoelectric polarization -> Z component

The difference between the processing results of the two surfaces is considered the charge response of the cantilever to the acceleration. A data fitting in MATLAB 2021 is conducted to extract the linear sensitivity.

The FEA simulation results in the table below are used in the data fitting. The data fitting result is shown in Figure S3. It exhibits a high linear relationship, indicating the validity of the derived model to certain extent.

| **Height (mm)** | **Width (mm)** | **Length (mm)** | **Fundamental resonant frequency (Hz)** | **Sensitivity (pC/g)** |
| --- | --- | --- | --- | --- |
| 0.05 | 2.5 | 2.5 | 492.96 | 2.48 |
| 0.05 | 3.0 | 2.5 | 494.18 | 2.97 |
| 0.05 | 3.5 | 2.5 | 495.09 | 3.46 |
| 0.05 | 2.5 | 5.0 | 208.83 | 9.27 |
| 0.05 | 3.0 | 5.0 | 208.85 | 11.12 |
| 0.05 | 3.5 | 5.0 | 206.73 | 12.93 |
| 0.05 | 2.5 | 7.5 | 110.96 | 21.17 |
| 0.05 | 3.0 | 7.5 | 114.44 | 25.43 |
| 0.05 | 3.5 | 7.5 | 115.14 | 29.74 |
| 0.1 | 2.5 | 2.5 | 1008.6 | 1.12 |
| 0.1 | 3.0 | 2.5 | 1012.6 | 1.35 |
| 0.1 | 3.5 | 2.5 | 1027.1 | 1.57 |
| 0.1 | 2.5 | 5.0 | 399.61 | 4.29 |
| 0.1 | 3.0 | 5.0 | 407.05 | 5.17 |
| 0.1 | 3.5 | 5.0 | 407.8 | 6.01 |
| 0.1 | 2.5 | 7.5 | 221.51 | 10.13 |
| 0.1 | 3.0 | 7.5 | 220.11 | 12.15 |
| 0.1 | 3.5 | 7.5 | 227.79 | 14.21 |

Table S1: FEA simulation results for different rectangular cantilevers with PVDF layers


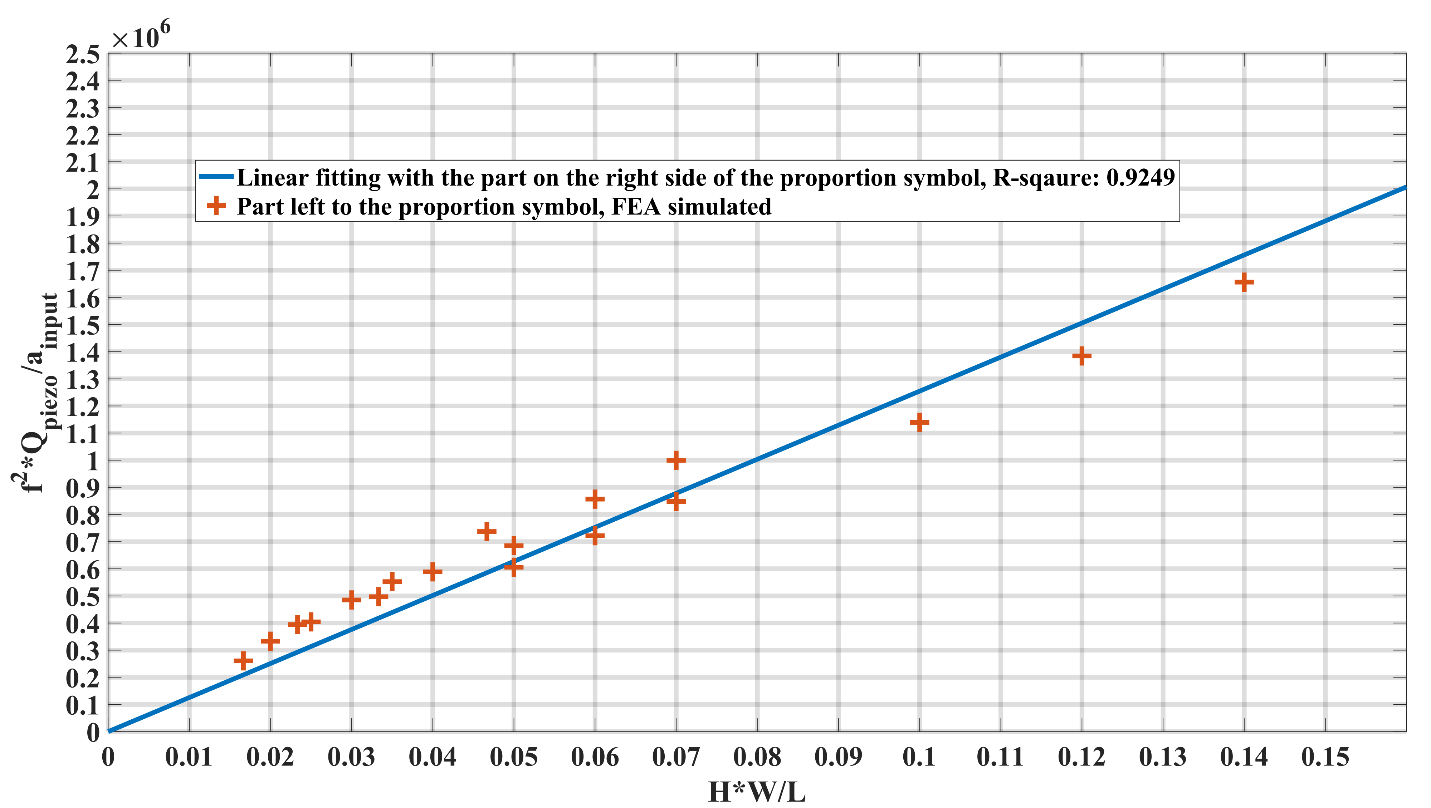


Figure S3 Data fitting between FEA simulated values and the geometric design parameters.

**Reference**

1 Volterra, E., Zachmanoglou, E. C. & Kolsky, H.

2 Budynas, R. G., Nisbett, J. K. & Shigley, J. E. *Shigley's mechanical engineering design*. Eleventh edn, (McGraw-Hill Education, 2020).

3 Polcawich, R. G. & Pulskamp, J. S. in *MEMS Materials and Processes Handbook* (eds Reza Ghodssi & Pinyen Lin) 273-353 (Springer US, 2011).

4 in *Inertial MEMS: Principles and Practice* (ed Volker Kempe) 283-363 (Cambridge University Press, 2011).
