# Supplementary material for: Polymeric piezoelectric accelerometers with high sensitivity, broad bandwidth, and low noise density for organic electronics and wearable microsystems
Source: Microsyst Nanoeng. 2024 May 15;10:61. doi: 10.1038/s41378-024-00704-6 (PMC11093978; doi:10.1038/s41378-024-00704-6)

**Supplement Material C5: Detailed measurement for Sample 5**

For all five samples, the mechanical resonant spectra were obtained from LDV measurements of the scanning points on the trapezoid cantilever sensing units. The measurements were saved as .svd files. In the PSV Viewer software, the average spectrum of all scanning points was extracted as the .txt files. They are read by Microsoft Excel and MATLAB to get the final results presented here and in the paper. As for the acceleration sensing tests, the readings on the oscilloscope were saved as .png files and presented here. The readings are based on the result of the math operation option: amplitude or the Y1-Y2 of the cursors in the oscilloscope.

# Sample 5

## Mechanical resonance measurement


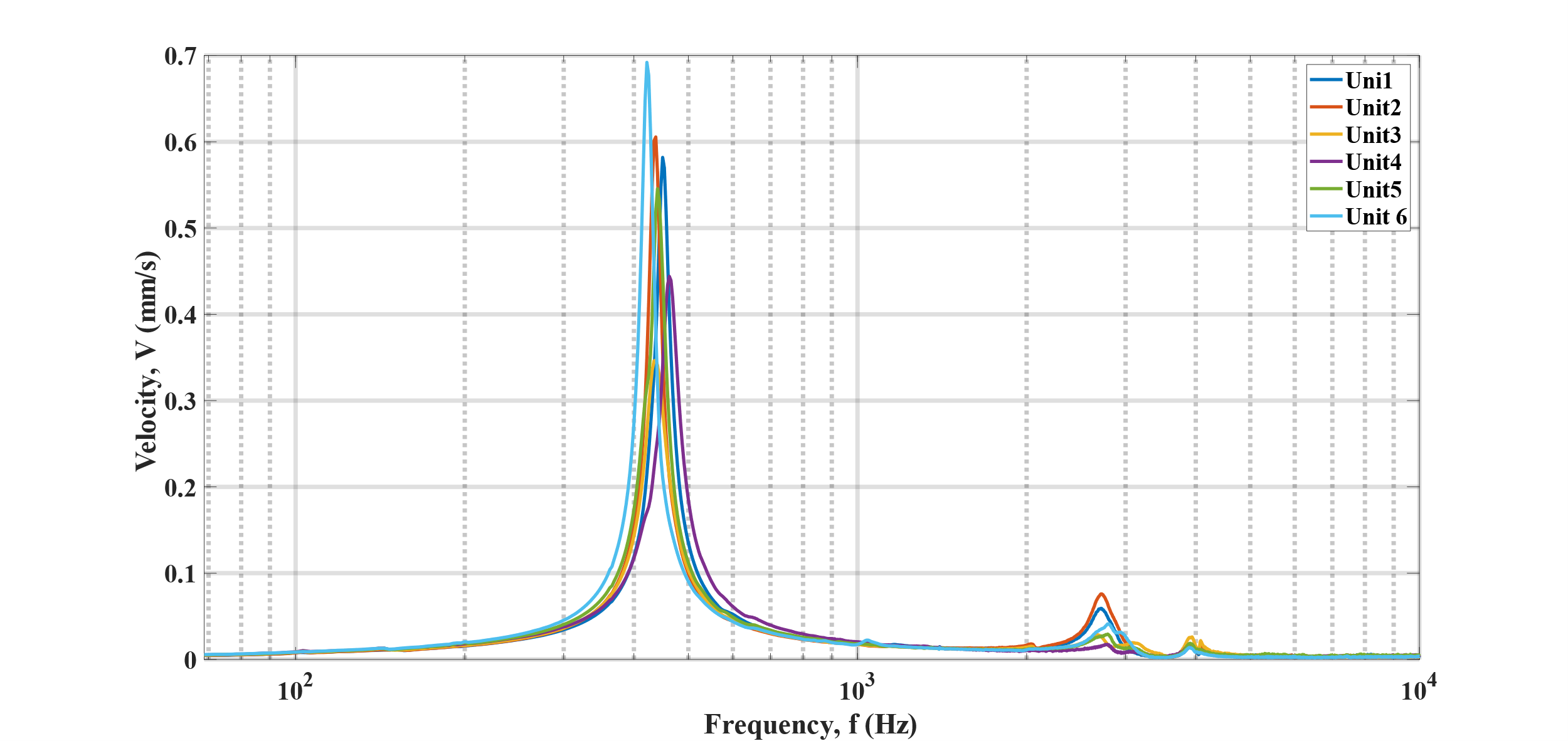


## Acceleration sensing test, frequency response, 50 Hz to 1000 Hz


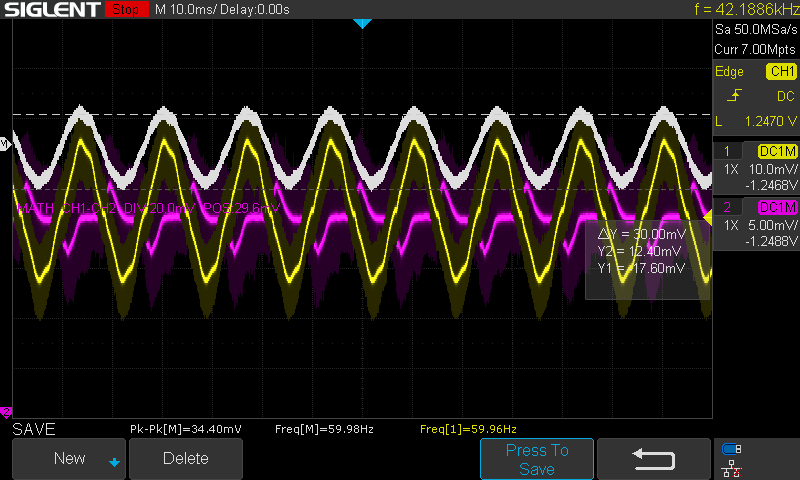

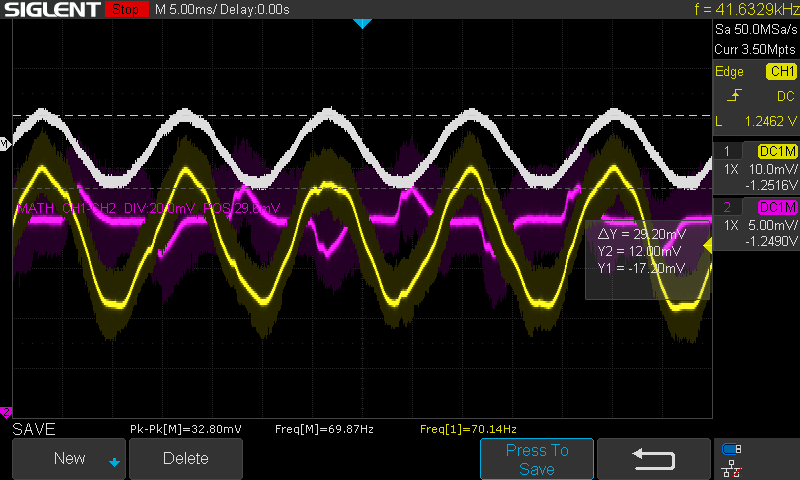

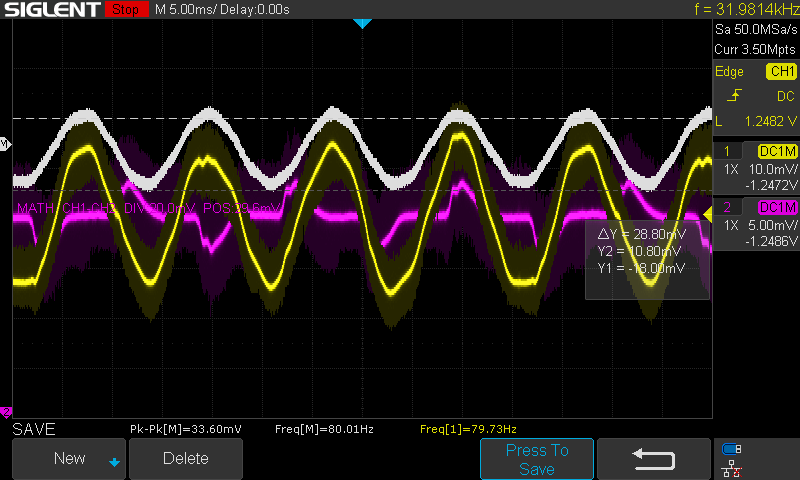

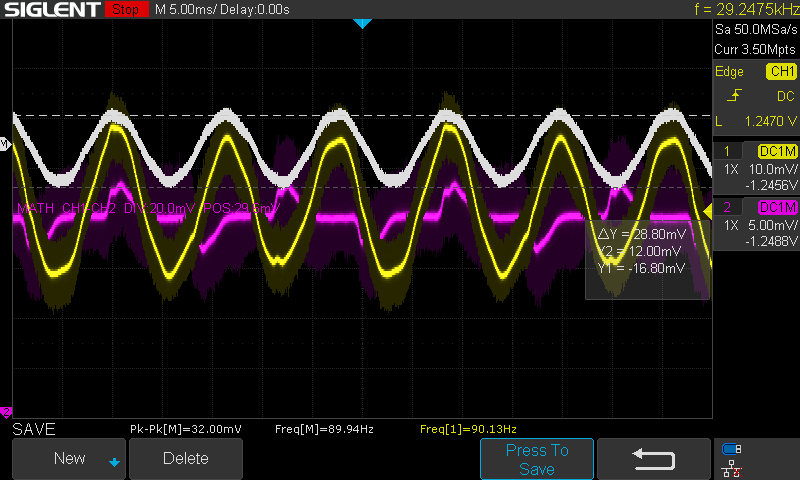

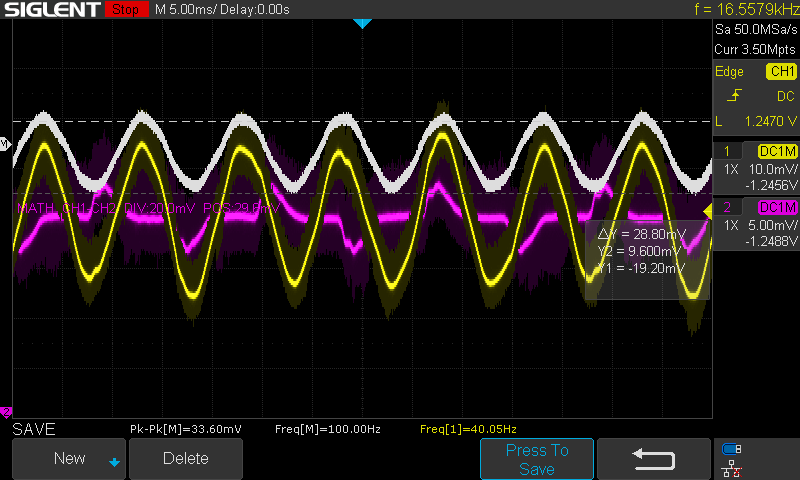

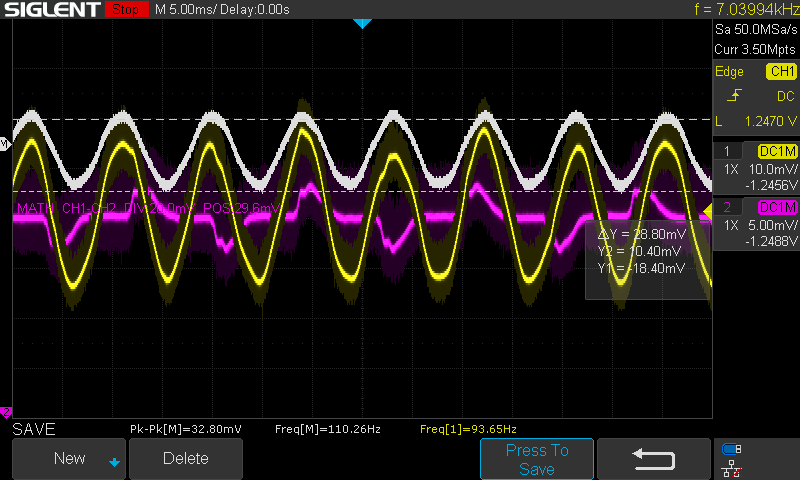

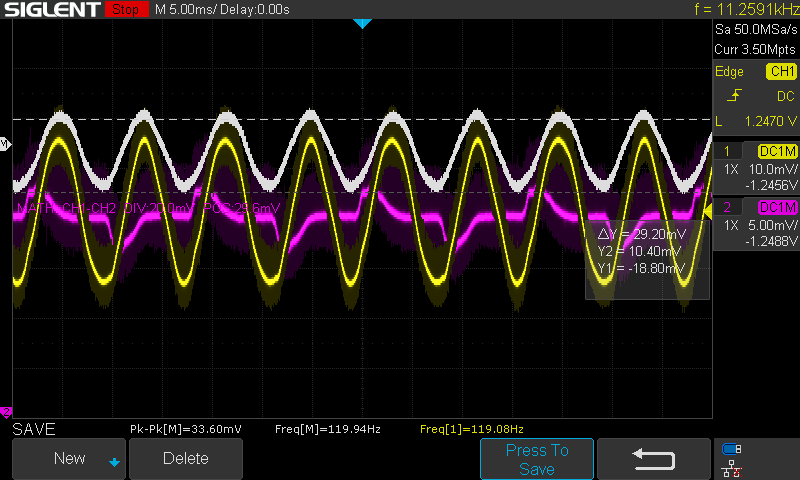

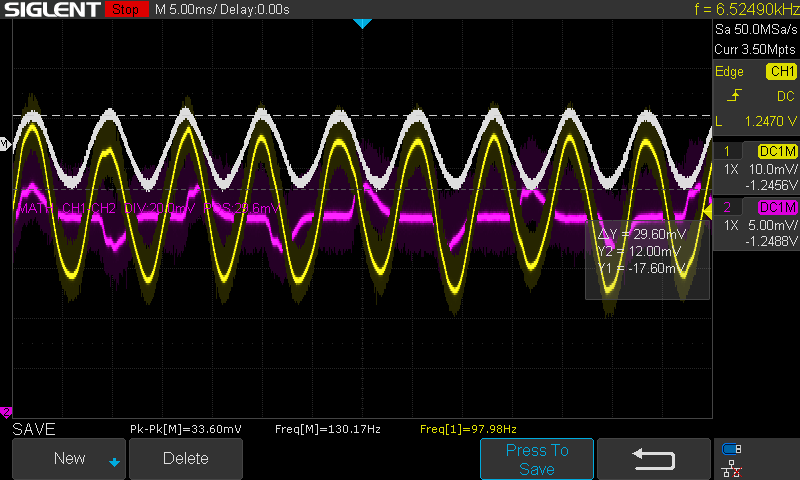

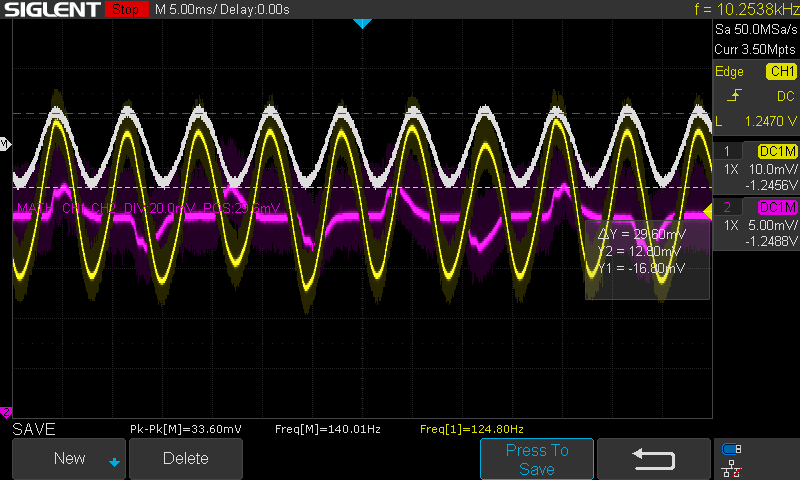

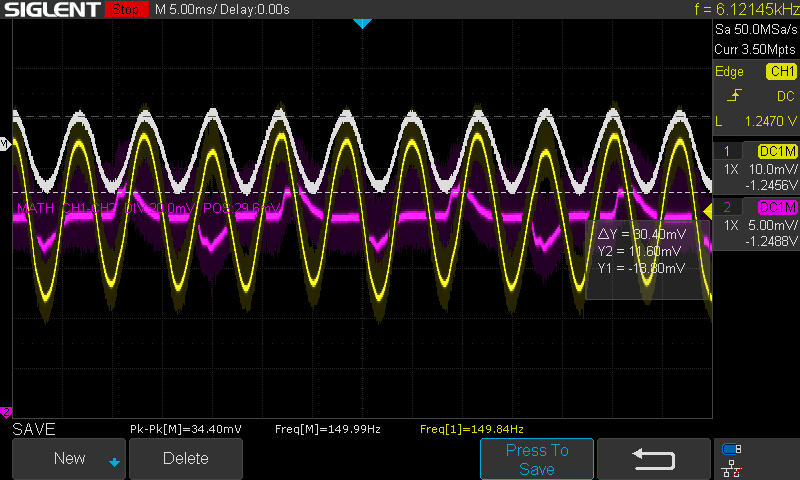

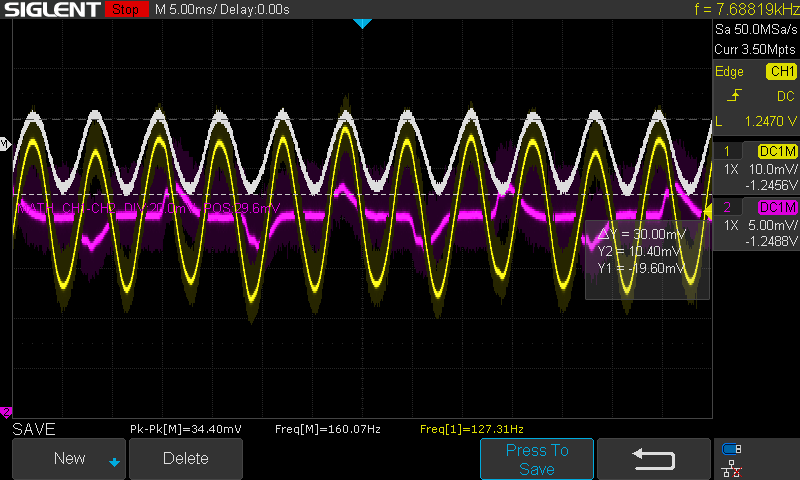

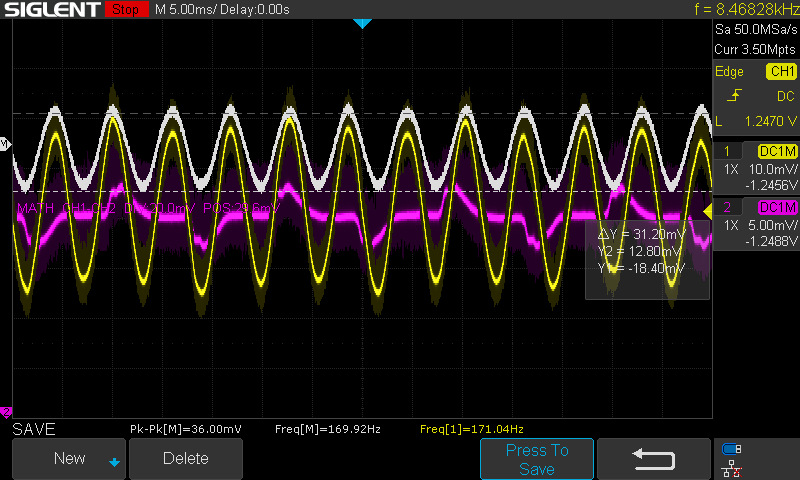

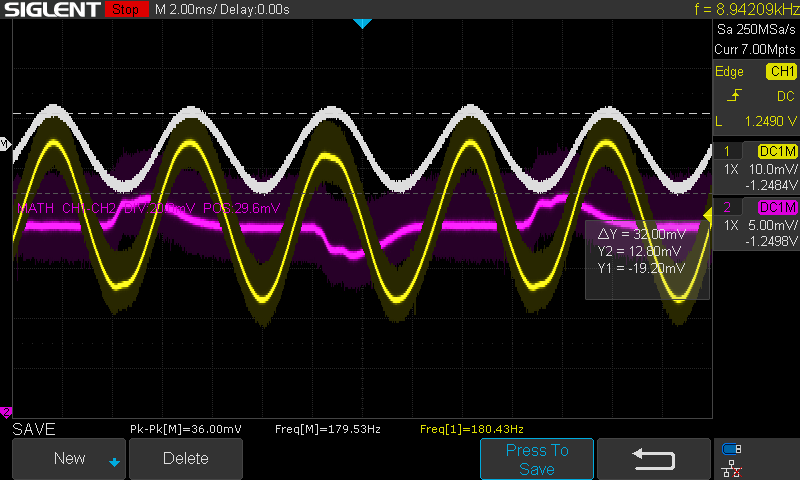

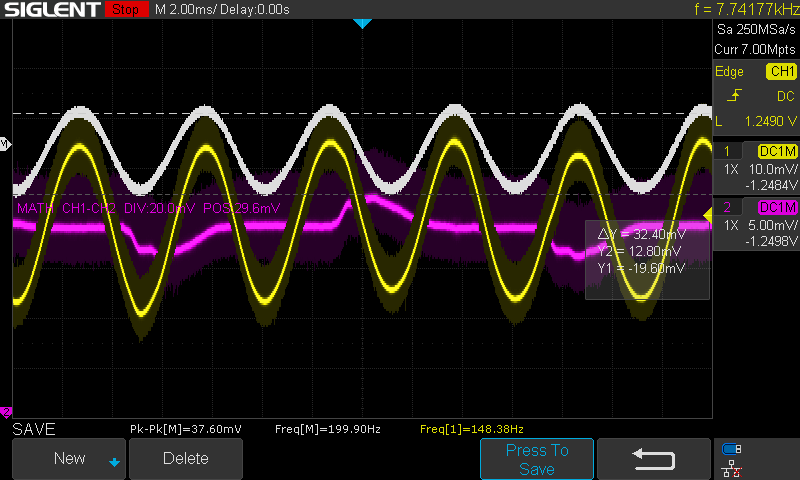

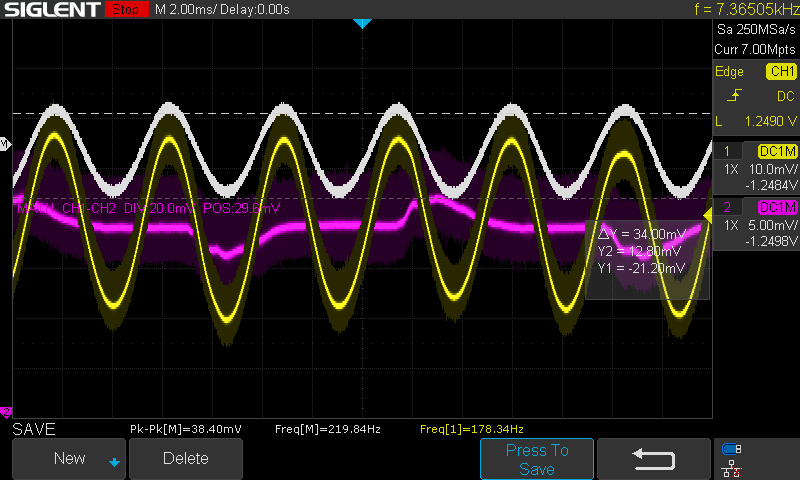

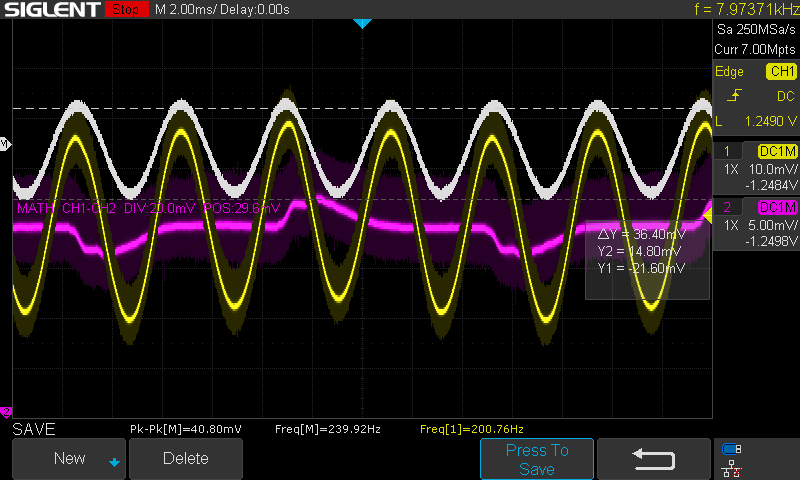

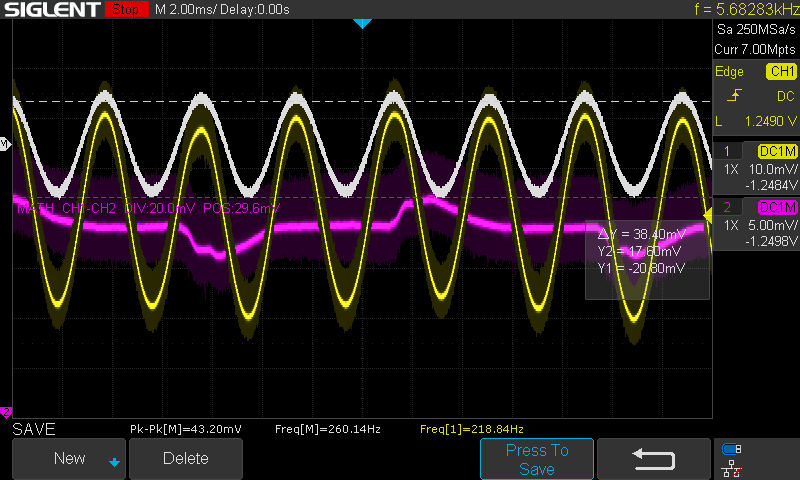

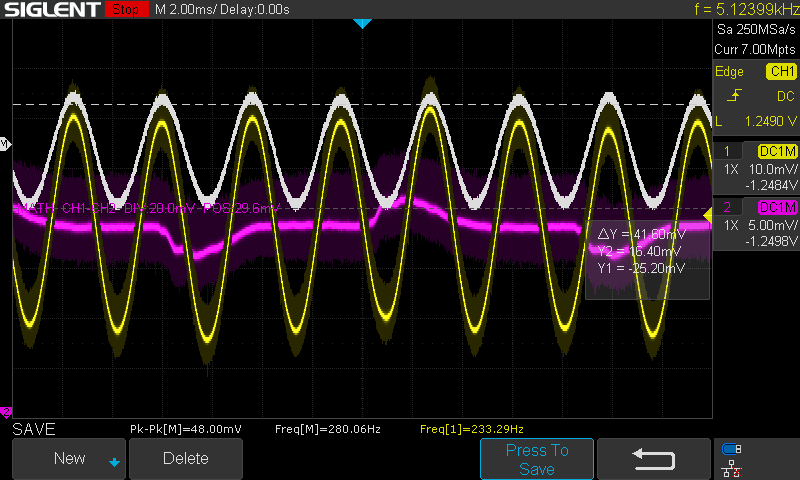

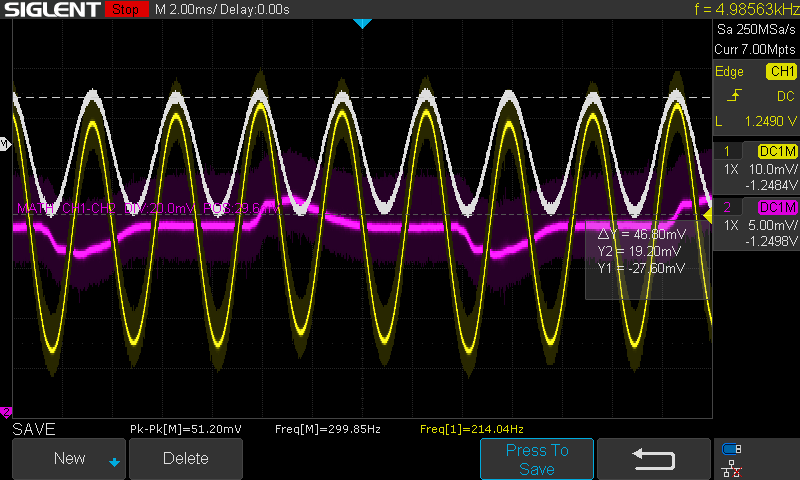

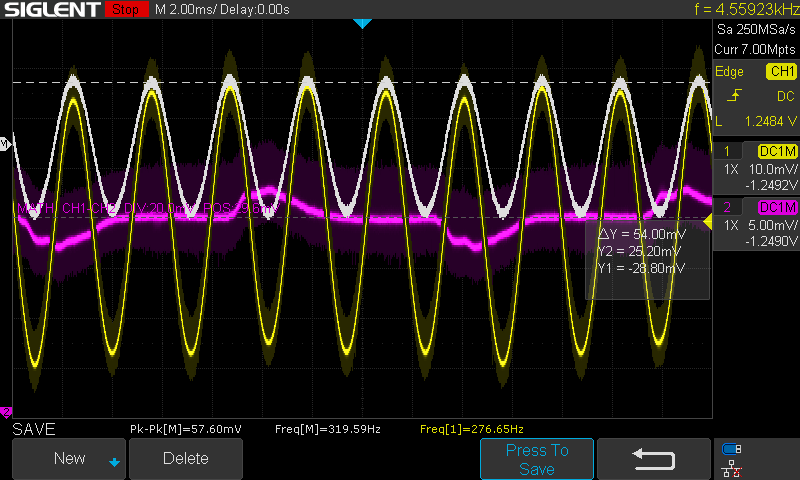

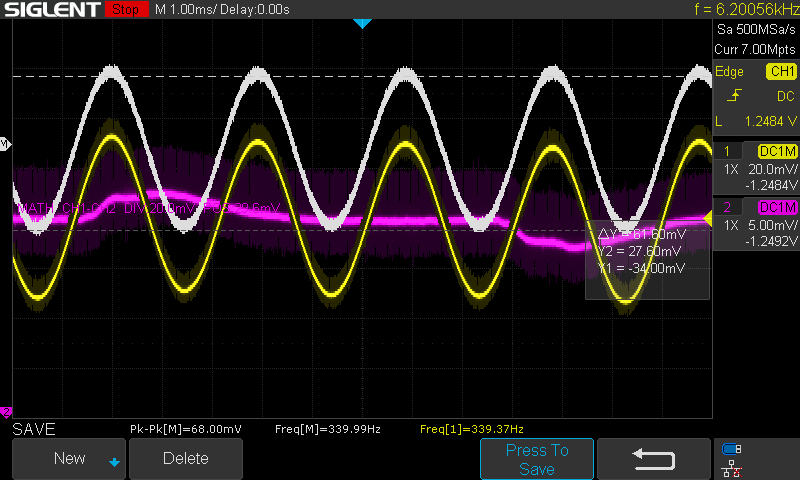

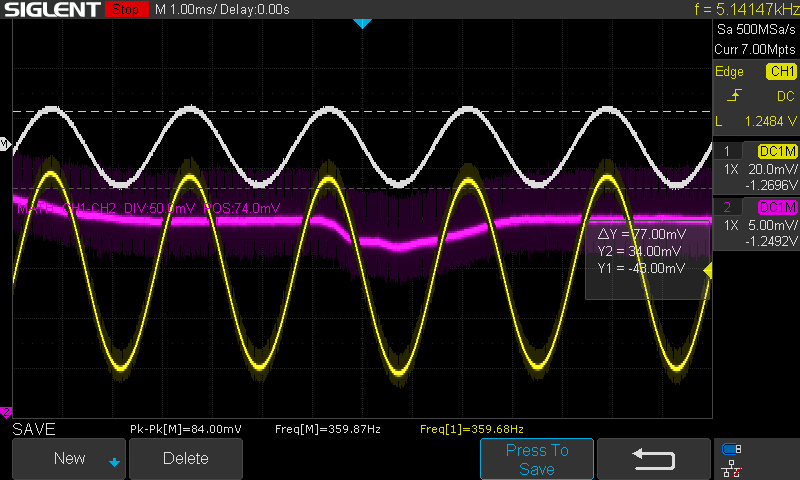

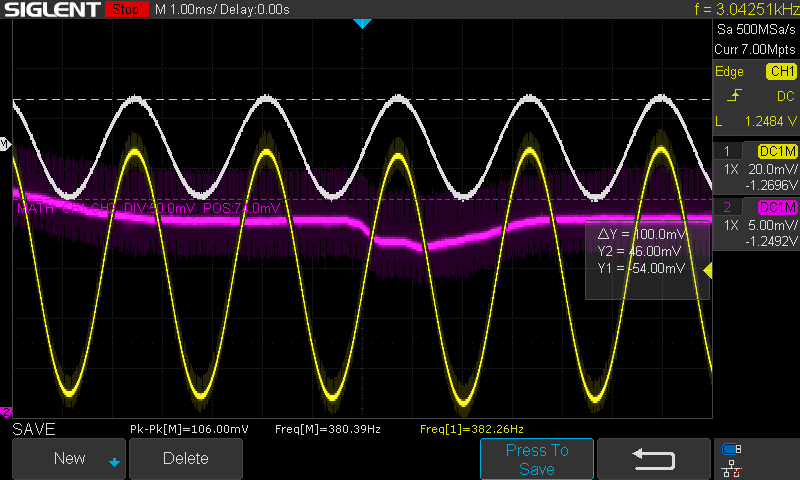

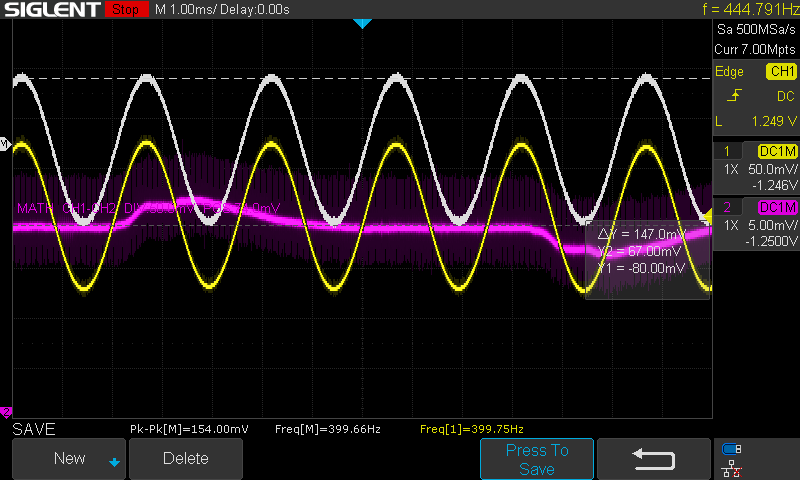

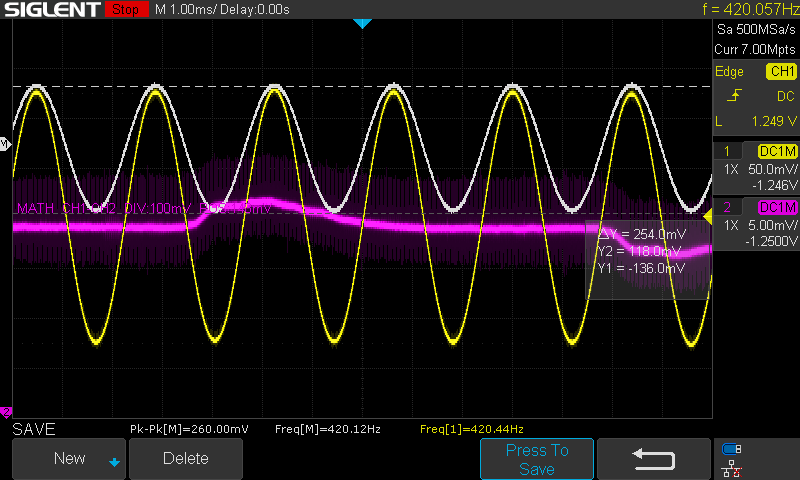

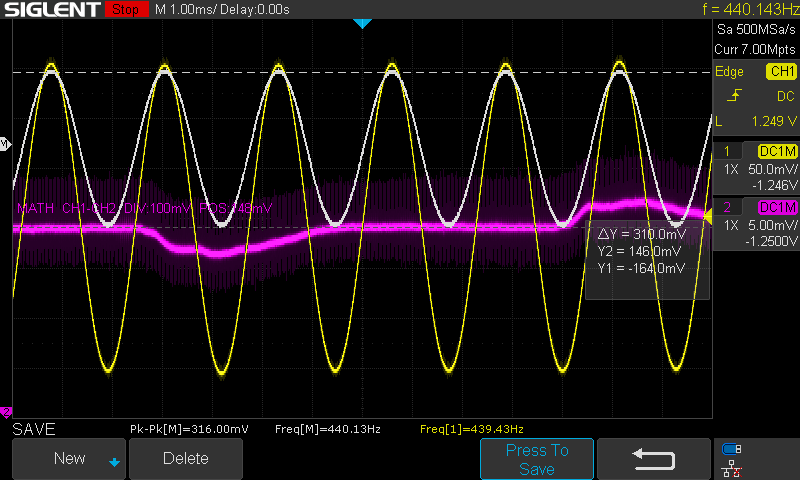

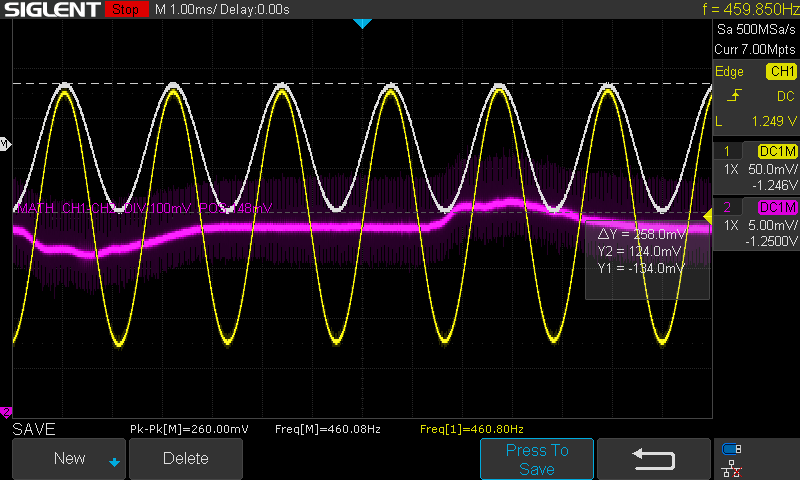

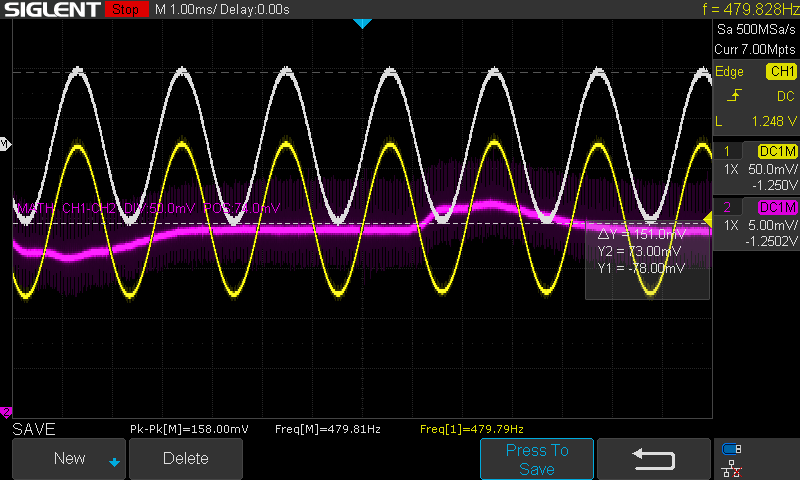

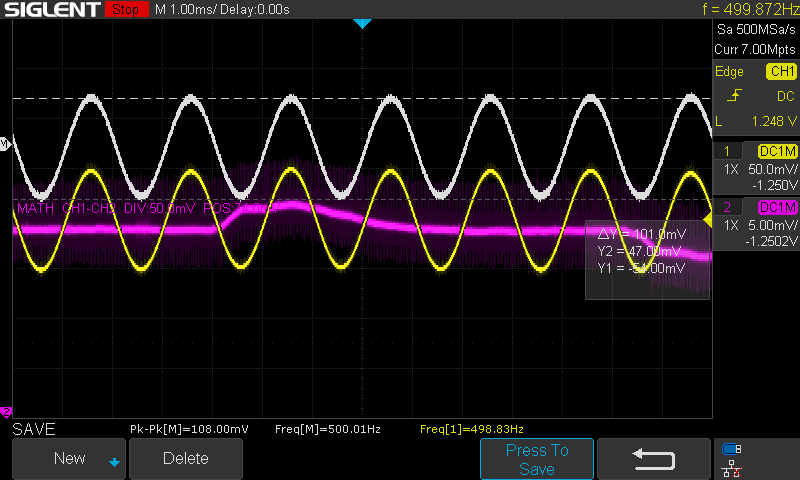

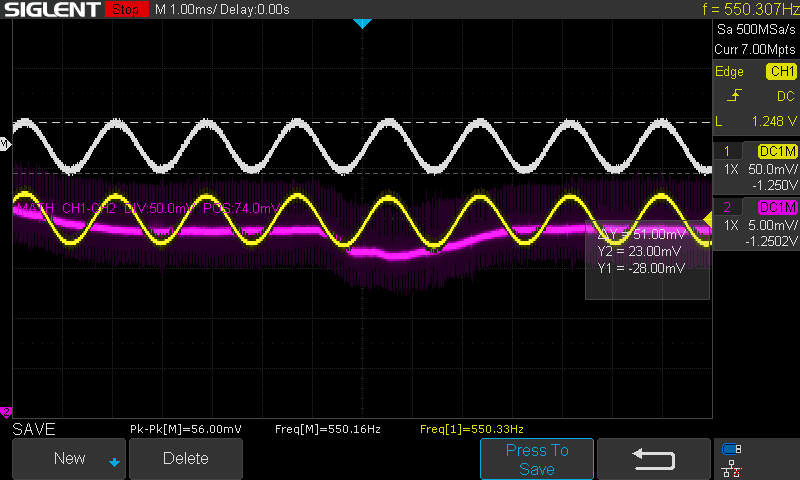

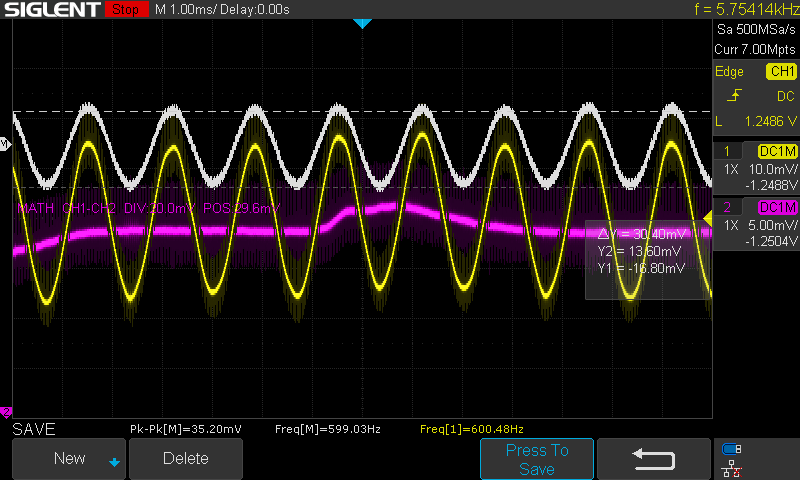

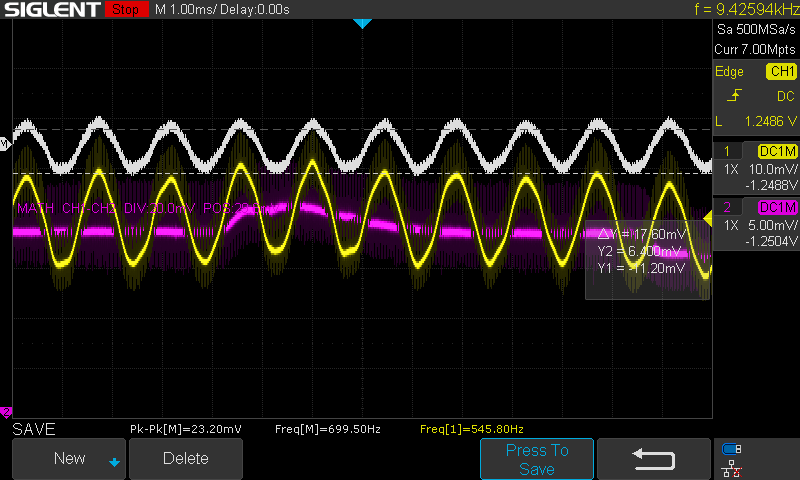

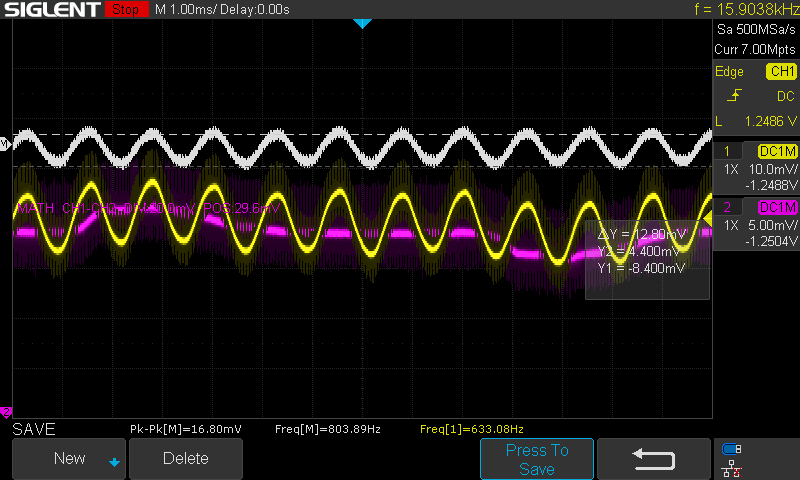

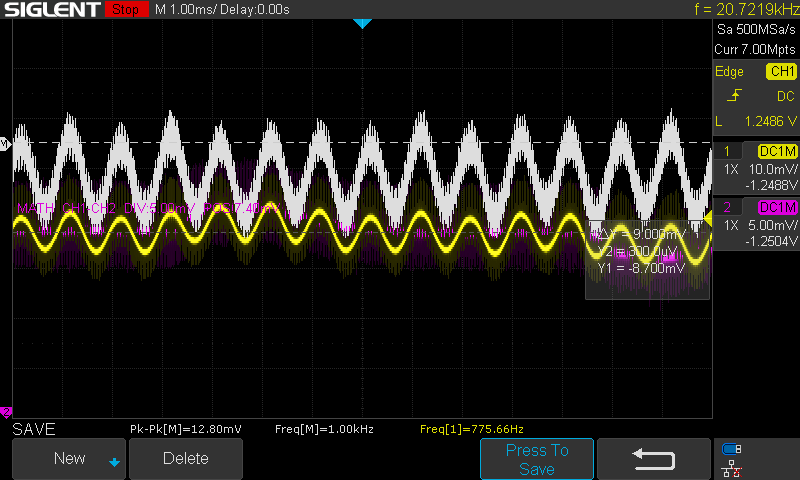


## Acceleration sensing test, flat-band sensitivity at 130 Hz, 0 to 10g


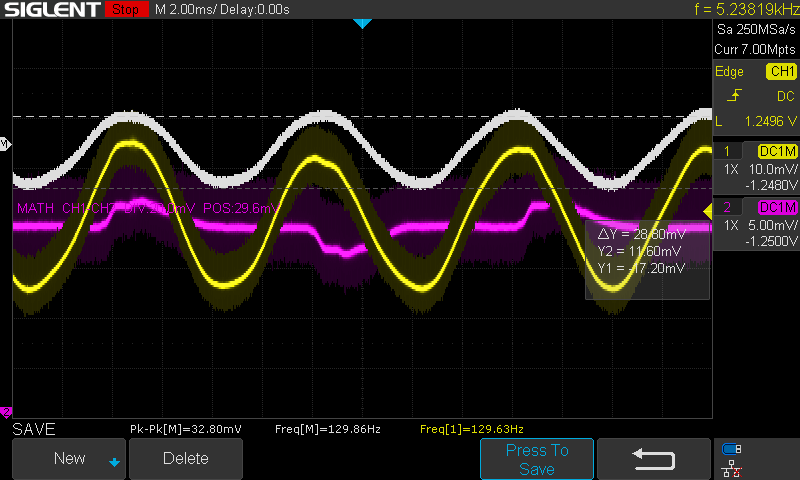

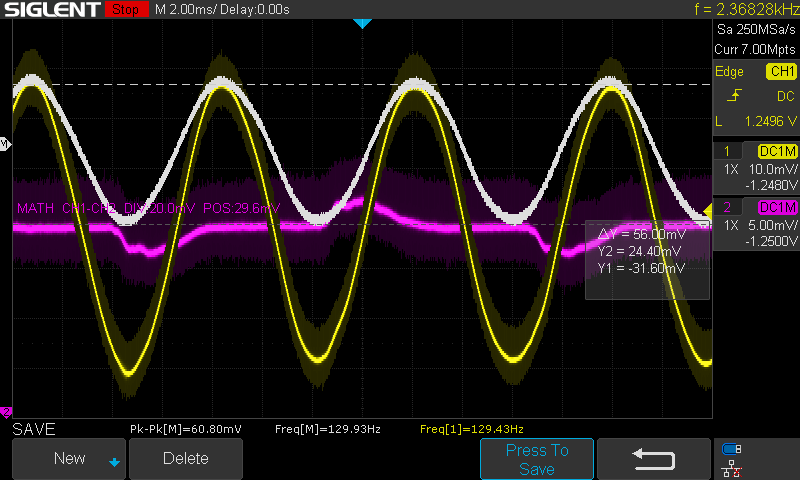

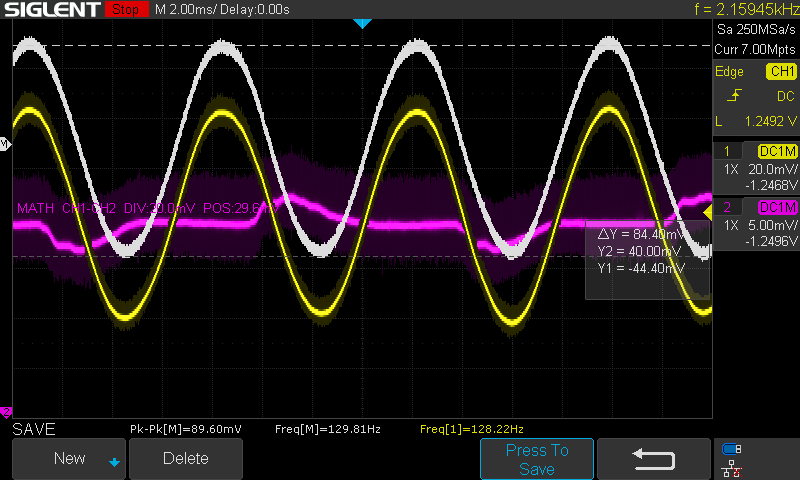

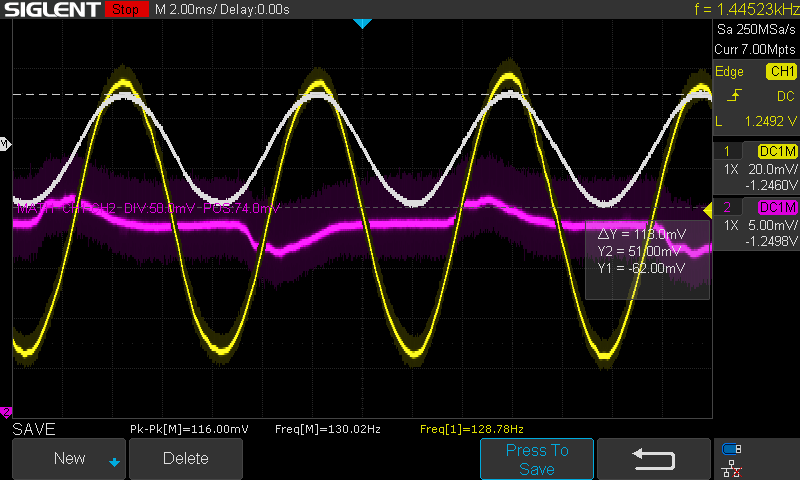

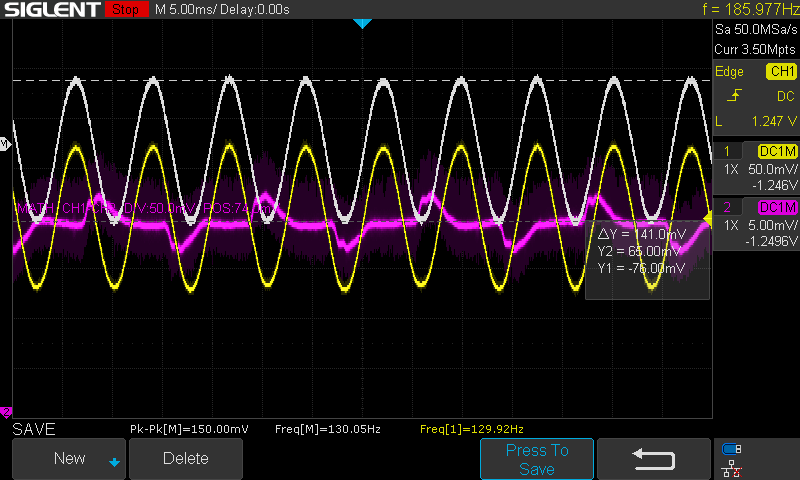

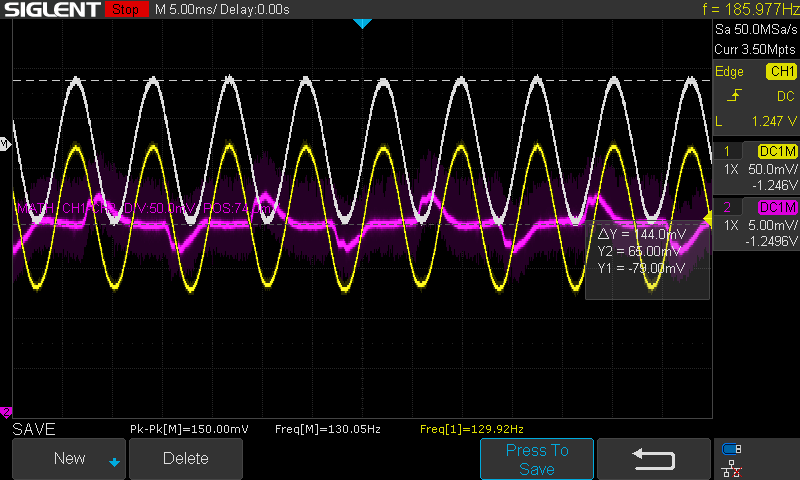

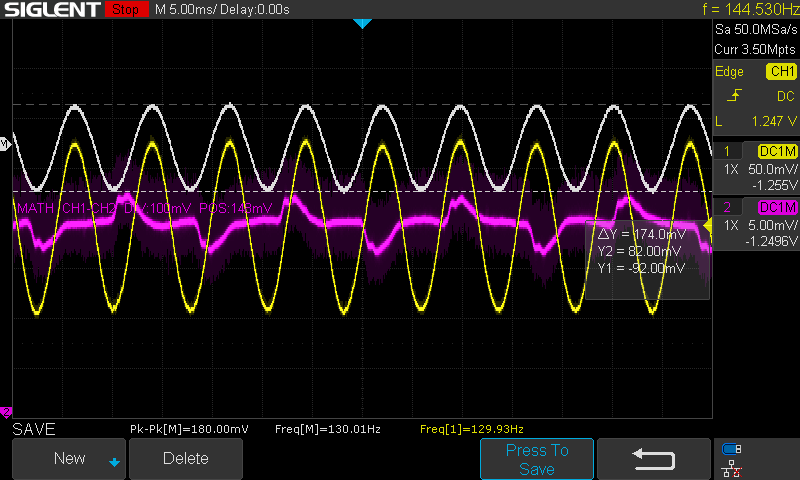

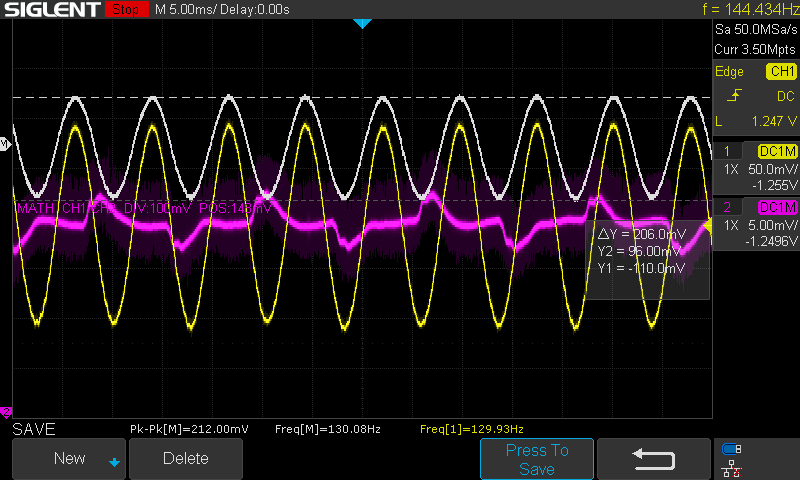

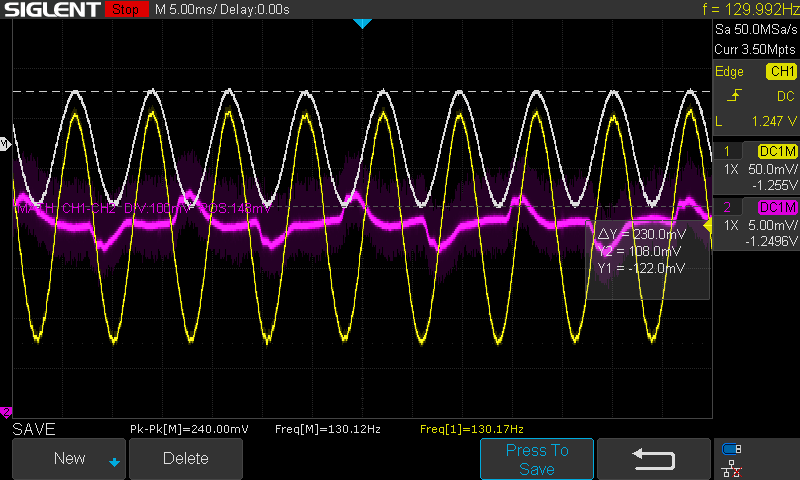

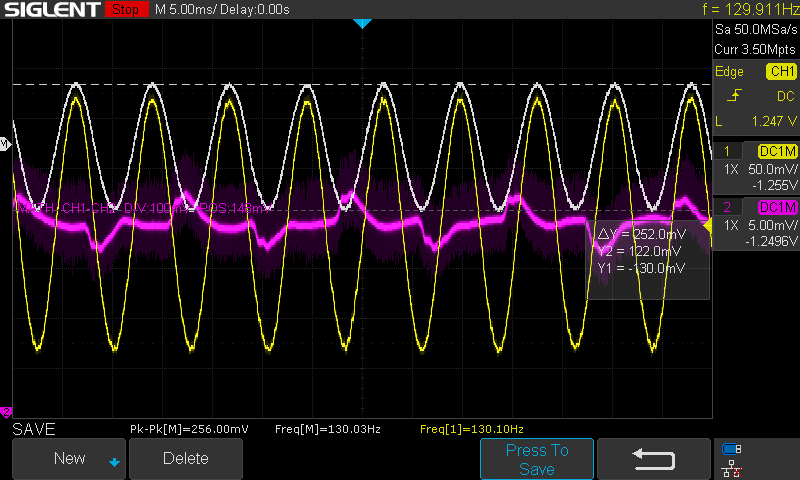

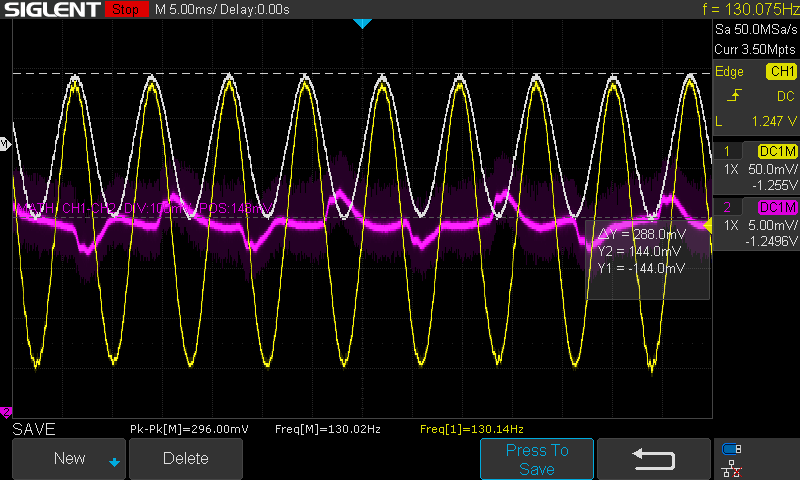

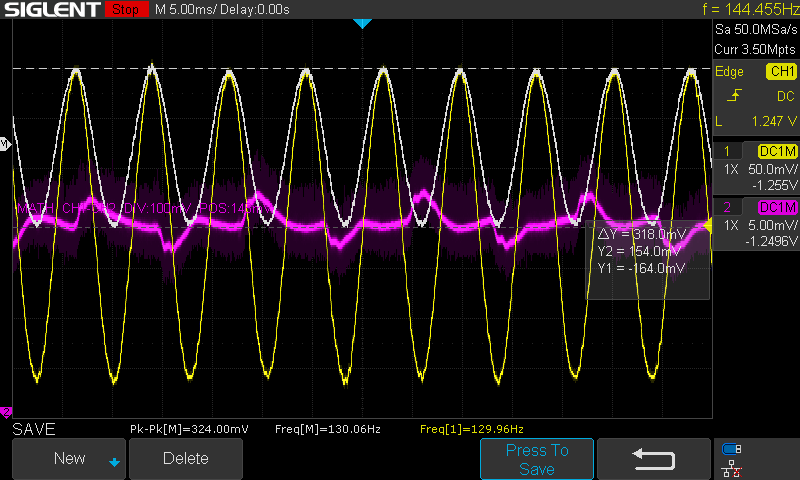

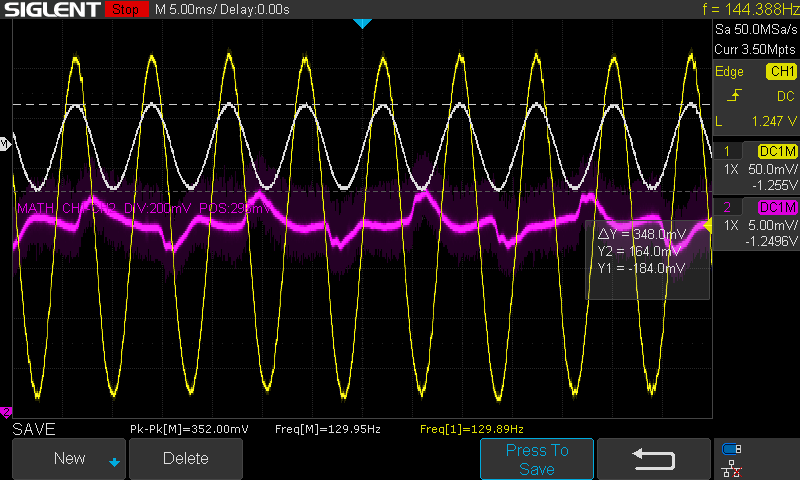

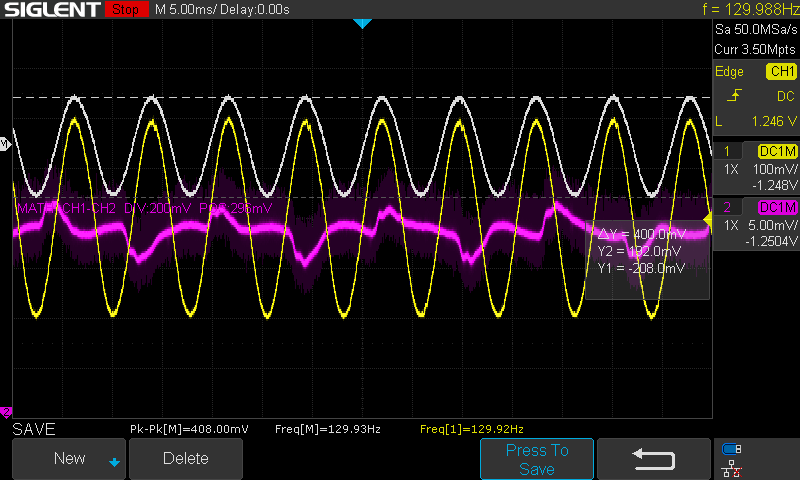

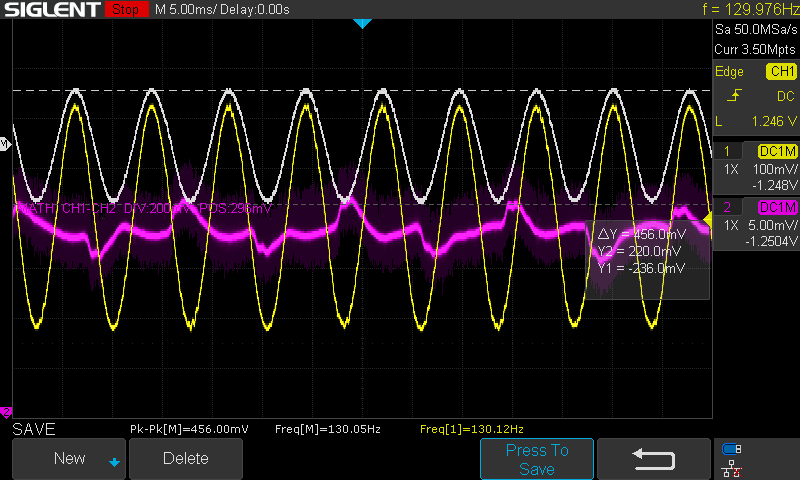

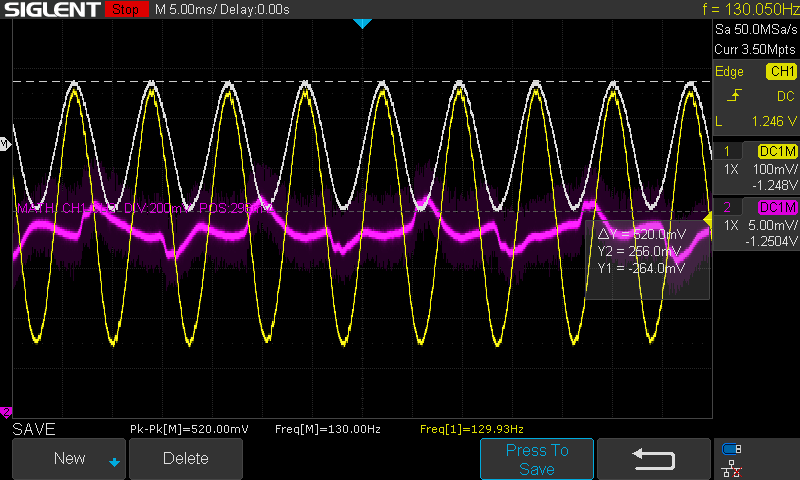

Supplement: Supplementary file 7 — Supplement material C5 Measurement result for Sample 5 [file 41378_2024_704_MOESM7_ESM.docx]
